# Functional Analysis of H^+^-Pumping Membrane-Bound Pyrophosphatase, ADP-Glucose Synthase, and Pyruvate Phosphate Dikinase as Pyrophosphate Sources in Clostridium thermocellum

**DOI:** 10.1128/aem.01857-21

**Published:** 2022-02-22

**Authors:** Teun Kuil, Shuen Hon, Johannes Yayo, Charles Foster, Giulia Ravagnan, Costas D. Maranas, Lee R. Lynd, Daniel G. Olson, Antonius J. A. van Maris

**Affiliations:** a Department of Industrial Biotechnology, School of Engineering Sciences in Chemistry, Biotechnology and Health, KTH Royal Institute of Technology, Stockholm, Sweden; b Thayer School of Engineering, Dartmouth College, Hanover, New Hampshire, USA; c Center for Bioenergy Innovation, Oak Ridge National Laboratory, Oak Ridge, Tennessee, USA; d Department of Chemical Engineering, The Pennsylvania State University, University Park, Pennsylvania, USA; University of Nebraska-Lincoln

**Keywords:** pyrophosphate, PP_i_, atypical glycolysis, H^+^-pumping membrane-bound pyrophosphatase, glycogen cycling, Ppdk, acetate cycling, functional annotation, *Clostridium thermocellum*, *Acetivibrio thermocellus*

## Abstract

The atypical glycolysis of Clostridium thermocellum is characterized by the use of pyrophosphate (PP_i_) as a phosphoryl donor for phosphofructokinase (Pfk) and pyruvate phosphate dikinase (Ppdk) reactions. Previously, biosynthetic PP_i_ was calculated to be stoichiometrically insufficient to drive glycolysis. This study investigates the role of a H^+^-pumping membrane-bound pyrophosphatase, glycogen cycling, a predicted Ppdk–malate shunt cycle, and acetate cycling in generating PP_i_. Knockout studies and enzyme assays confirmed that *clo1313_0823* encodes a membrane-bound pyrophosphatase. Additionally, *clo1313_0717-0718* was confirmed to encode ADP-glucose synthase by knockouts, glycogen measurements in *C. thermocellum*, and heterologous expression in Escherichia coli. Unexpectedly, individually targeted gene deletions of the four putative PP_i_ sources did not have a significant phenotypic effect. Although combinatorial deletion of all four putative PP_i_ sources reduced the growth rate by 22% (0.30 ± 0.01 h^−1^) and the biomass yield by 38% (0.18 ± 0.00 g_biomass_ g_substrate_^−1^), this change was much smaller than what would be expected for stoichiometrically essential PP_i_-supplying mechanisms. Growth-arrested cells of the quadruple knockout readily fermented cellobiose, indicating that the unknown PP_i_-supplying mechanisms are independent of biosynthesis. An alternative hypothesis that ATP-dependent Pfk activity circumvents a need for PP_i_ altogether was falsified by enzyme assays, heterologous expression of candidate genes, and whole-genome sequencing. As a secondary outcome, enzymatic assays confirmed functional annotation of *clo1313_1832* as ATP- and GTP-dependent fructokinase. These results indicate that the four investigated PP_i_ sources individually and combined play no significant PP_i_-supplying role, and the true source(s) of PP_i_, or alternative phosphorylating mechanisms, that drive(s) glycolysis in *C. thermocellum* remain(s) elusive.

**IMPORTANCE** Increased understanding of the central metabolism of *C. thermocellum* is important from a fundamental as well as from a sustainability and industrial perspective. In addition to showing that H^+^-pumping membrane-bound PPase, glycogen cycling, a Ppdk–malate shunt cycle, and acetate cycling are not significant sources of PP_i_ supply, this study adds functional annotation of four genes and availability of an updated PP_i_ stoichiometry from biosynthesis to the scientific domain. Together, this aids future metabolic engineering attempts aimed to improve *C. thermocellum* as a cell factory for sustainable and efficient production of ethanol from lignocellulosic material through consolidated bioprocessing with minimal pretreatment. Getting closer to elucidating the elusive source of PP_i_, or alternative phosphorylating mechanisms, for the atypical glycolysis is itself of fundamental importance. Additionally, the findings of this study directly contribute to investigations into trade-offs between thermodynamic driving force versus energy yield of PP_i_- and ATP-dependent glycolysis.

## INTRODUCTION

The anaerobic cellulolytic thermophile Clostridium thermocellum (also named Ruminiclostridium thermocellum, Hungateiclostridium thermocellum, and Acetivibrio thermocellus [1]) is a promising candidate organism for consolidated bioprocessing of lignocellulosic biomass into ethanol (2–4). Despite several metabolic engineering attempts aimed at increasing the ethanol yield and titer, which have resulted in a yield that is 75% of the theoretical maximum (5) and a maximum ethanol titer of 30 g/L (6), further improvements are necessary for industrial implementation (7). Increased understanding of the central metabolism of *C. thermocellum* would help guide such metabolic engineering strategies.

**FIG 1:**
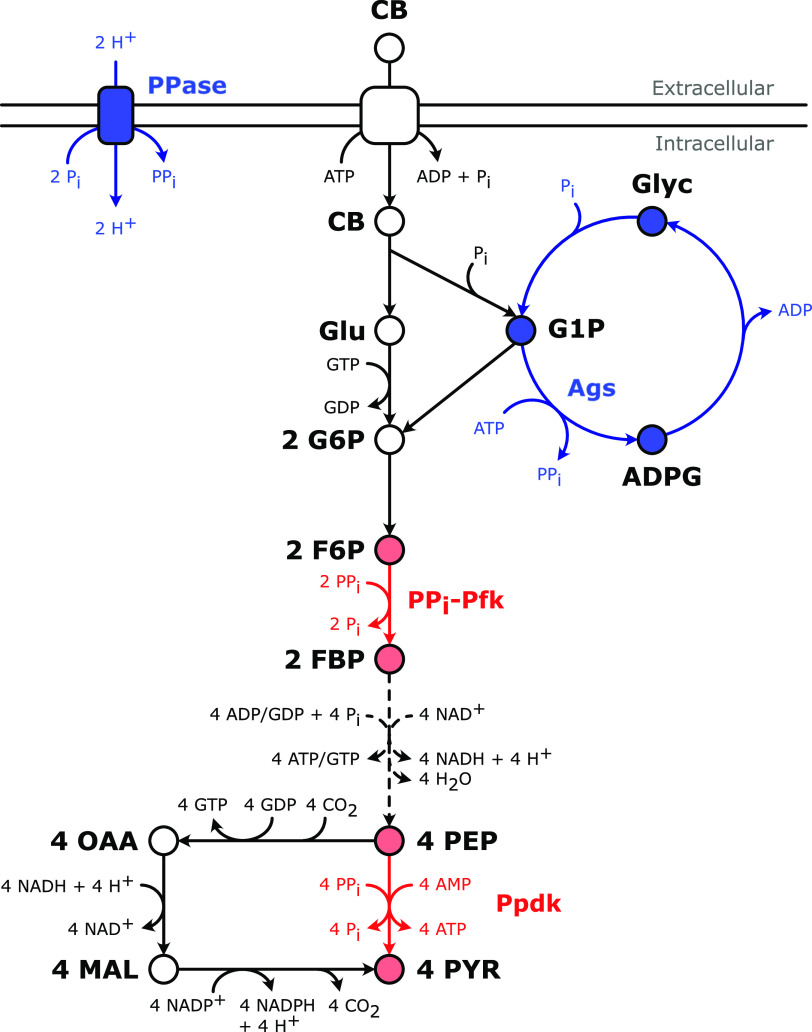
PP_i_-dependent glycolysis of Clostridium thermocellum. PP_i_ is consumed by PP_i_-dependent phosphofructokinase (PP_i_-Pfk) and pyruvate phosphate dikinase (Ppdk) (indicated in red) and may be formed by the proton-pumping membrane-bound pyrophosphatase (PPase) and glycogen cycling (indicated in blue), with ADP-glucose synthase (Ags) as the key enzyme. The H^+^/PP_i_ stoichiometry of the PPase is based on previous estimations (2). Solid arrows represent a single reaction; dashed arrows represent lumped reactions. Abbreviations: ADPG, ADP-glucose; CB, cellobiose; F6P, fructose-6-phosphate; FBP, fructose-1,6-bisphosphate; G1P, glucose-1-phosphate; G6P, glucose-6-phosphate; Glu, glucose; Glyc, glycogen; Mal, malate; OAA, oxaloacetate; PEP, phosphoenolpyruvate; PYR, pyruvate.

One of the remarkable features of the central metabolism of *C. thermocellum* is the key role of pyrophosphate (PP_i_) in its atypical glycolysis. In contrast to the canonical Embden-Meyerhof-Parnas glycolytic pathway, which uses an ATP-dependent phosphofructokinase (Pfk) and pyruvate kinase, *C. thermocellum* uses a PP_i_-dependent Pfk and pyruvate phosphate dikinase (Ppdk) (8, 9) (Fig. 1). As an alternative for formation of pyruvate through the Ppdk reaction, pyruvate can also be formed from phosphoenolpyruvate (PEP) through the malate shunt, consisting of three sequential reactions catalyzed by PEP carboxykinase, malate dehydrogenase, and malic enzyme (8, 10) (Fig. 1). The glycolysis of *C. thermocellum* not only has PP_i_ and ATP dependent steps but also uses GTP for the hexokinase and PEP carboxykinase reactions, whereas phosphoglycerate kinase is equally active with ADP or GDP (9). Assuming that the PEP-to-pyruvate conversion goes solely through Ppdk, ATP and GTP are energetically equivalent (written as ATP_eq_), and AMP, ADP, and ATP are balanced by adenylate kinase (which means Ppdk and adenylate kinase together result in net 8 ADP being phosphorylated to 8 ATP; Fig. 1), glycolysis starting from cellobiose would have a net stoichiometry according to equation 1:

(1) cellobiose + 6  PPi + 10 ADPeq + 4  NAD+→4  pyruvate + 10  ATPeq + 2  Pi + 4  NADH + 4  H+

Given that cellobiose consists of two glucose equivalents, this can be written as

(2) glucoseeq + 3 PPi + 5 ADPeq + 2 NAD+→2 pyruvate + 5 ATPeq + Pi + 2 NADH + 2  H+

Hence, depending on the source of PP_i_, glycolysis could maximally yield 5 ATP equivalents per glucose equivalent. On the more preferred longer soluble cellulose-hydrolysis products (i.e., cellodextrins) of length *n*, such as cellotetraose (*n *= 4), *C. thermocellum* can conserve additional ATP due to the phosphoroclastic cleavage of cellodextrin to glucose-1-phosphate and free cellodextrin of length *n* – 1, catalyzed by cellodextrin phosphorylase (2). Consistent with this unusually high ATP gain, glycolysis in *C. thermocellum* has been shown to be much more reversible than that in several other bacteria growing anaerobically (11, 12), and it has been hypothesized that this contributes to the relatively low ethanol titers produced in engineered strains thus far (11).

PP_i_ is produced as a by-product of energy-requiring biosynthetic reactions (i.e., during DNA, RNA, protein, polysaccharide, and lipid synthesis) and is, in many organisms, hydrolyzed to orthophosphate (P_i_) by soluble inorganic pyrophosphatases (PPase) (13, 14). This dissipates the energy of the phosphoanhydride bond, prevents build-up of PP_i_, and increases the thermodynamic driving force for biosynthetic reactions (15). Most microorganisms that rely on PP_i_-dependent glycolysis, such as *C. thermocellum*, do not have such a soluble PPase (16). Instead, they can conserve the energy stored in the phosphoanhydride bond by recycling the biosynthetically generated PP_i_ in glycolysis, which hypothetically results in a net increase of the ATP yield compared to a conventional ATP-dependent glycolysis (14, 16, 17) (equation 2). However, subsequent model-based calculations estimated that the amount of PP_i_ generated from biosynthetic reactions is far from enough to satisfy the PP_i_ requirement in glycolysis (9). Hence, additional mechanisms to generate PP_i_ must be operative to be consistent with the current understanding of glycolysis in *C. thermocellum*.

Two possible mechanisms that have been postulated include the use of a H^+^-pumping membrane-bound pyrophosphatase and the use of glycogen cycling in which glycogen is simultaneously formed and degraded (9, 18) (Fig. 1). In Rhodospirillum rubrum and Arabidopsis thaliana, a H^+^-pumping membrane-bound PPase couples the energy stored in the proton gradient to formation of PP_i_ from 2 P_i_ (19–21). If, in *C. thermocellum*, this reaction indeed operates in the reverse PP_i_-generating direction, 1 mol ATP might drive synthesis of 2 mol PP_i_ based on previous estimations of the H^+^/PP_i_ stoichiometry of the PPase and the ATP/H^+^ stoichiometry of the ATPase (2). This would result in a net ATP yield of 3.5 ATP per mol glucose equivalent (equation 2). Although a previous study did not observe a phenotype upon knockout of a gene putatively encoding the membrane-bound PPase, it was not shown whether membrane-bound PPase activity was indeed eliminated (18). A second proposed mechanism, glycogen cycling, starts with the key enzyme ADP-glucose synthase catalyzing the reaction from glucose-1-P and ATP to ADP-glucose and PP_i_ (Fig. 1). Glycogen is subsequently formed from ADP-glucose and degraded again to glucose-1-P. Overall, this cycle would form one PP_i_ and ADP at the expense of one ATP and P_i_. If this mechanism would be solely responsible for PP_i_ supply, the net ATP yield will be 2 mol ATP per mol glucose equivalent (equation 2). Glycogen cycling has been observed before in anaerobic cellulolytic bacteria harboring PP_i_-dependent glycolysis (22–24); however, the role of such cycling in supplying PP_i_ has not yet been experimentally verified.

The aim of the present study is to investigate possible PP_i_-generating mechanisms in the central metabolic pathways of *C. thermocellum*. To this end, single knockout strains of two previously suggested and two newly identified candidate PP_i_-supplying routes were constructed, and the impact of the deletions was quantitatively analyzed in batch serum bottle cultures. Functional annotation of targeted genes and confirmation of their deletion was done through enzyme assays or glycogen measurements. To assess possible complementarity of the mechanisms, double, triple, and quadruple knockout strains were constructed and analyzed. Whole-genome sequencing was performed to identify possible secondary mutations. Finally, growth arrest studies of the constructed strains were performed to evaluate the role of biosynthetically generated PP_i_ in these strains.

## RESULTS

### Theoretical analysis of the pyrophosphate stoichiometry of biosynthesis.

**FIG 2:**
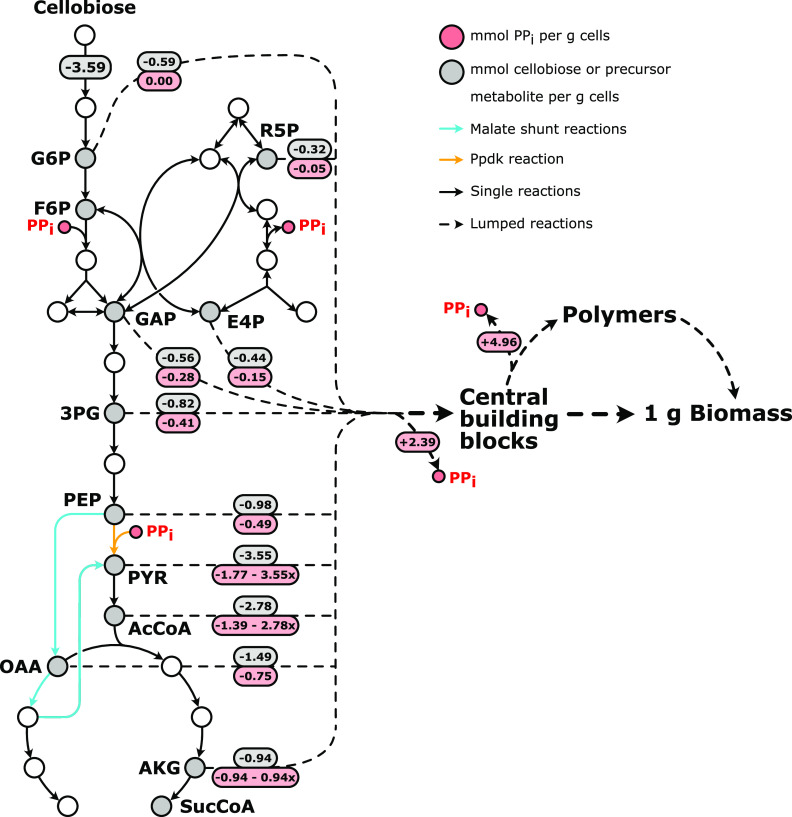
Theoretical pyrophosphate and carbon amounts (in mmol per g cells) needed for biomass formation from precursor metabolites for wild-type *C. thermocellum* grown on cellobiose. The PP_i_ and carbon fluxes are determined from the stoichiometric analysis of biosynthesis using the macromolecular composition of aerobically grown glucose-limited chemostat cultures of B. subtilis (Table 1). The parameter *x* (in the red boxes) describes the flux distribution between the Ppdk reaction and the malate shunt (*x* = 1, the PEP-to-pyruvate conversion solely goes through Ppdk; *x* = 0, the PEP-to-pyruvate conversion solely goes through the malate shunt). Numbers in gray boxes represent the amounts of cellobiose or precursor metabolites needed for biomass formation in mmol g*_x_*^−1^; numbers in red boxes represent the PP_i_ amounts produced or consumed per precursor metabolite needed for biomass formation in mmol g*_x_*^−1^. Gray circles represent precursor metabolites; red circles represent PP_i_; white circles represent nonprecursor metabolites. Solid arrows represent a single reaction; dashed arrows represent lumped reactions. Cyan arrows represent the malate shunt; the orange arrow represents the Ppdk reaction. For the PP_i_ stoichiometry of E4P and R5P, the nonoxidative pentose-phosphate pathway as proposed by Koendjbiharie et al. (60) is used. Abbreviations: 3PG, 3-phosphoglycerate; AcCoA, acetyl-coenzyme A; AKG, α-ketoglutarate; E4P, erythrose-4-phosphate; F6P, fructose-6-phosphate; G6P, glucose-6-phosphate; GAP, glyceraldehyde-3-phosphate; OAA, oxaloacetate; PEP, phosphoenolpyruvate; PYR, pyruvate; R5P, ribose-5-phosphate; SucCoA, succinyl-coenzyme A.

Previous studies on the glycolytic PP_i_ requirement of cellobiose-grown *C. thermocellum* cultures used an Escherichia coli metabolic network model (25) to estimate the amount of PP_i_ generated during biosynthesis (9, 18). This model predicted that approximately 11 mmol PP_i_ is produced per g biomass during biosynthesis, an amount that is not corrected for the anabolic PP_i_ requirement that occurs in *C. thermocellum* when the carbon flux required for assembling the building blocks for biosynthesis involves the PP_i_-consuming Pfk and Ppdk reactions. Zhou et al. (9) partially accounted for this by including the assumption that all carbon used for biosynthesis passes through the Pfk reaction, which still resulted in a predicted net biosynthetic PP_i_ formation of 3.6 mmol PP_i_ per g biomass. However, as Ppdk activity was not yet detected, this prediction did not include the PP_i_ consumption in lower glycolysis and therefore still overestimates the net biosynthetic PP_i_ stoichiometry. Considering that the Ppdk reaction likely carries a large part of the PEP-to-pyruvate flux (67% according to Olson et al. [8]), it is possible that in some cases biomass formation actually requires a net input of PP_i_ per gram of biomass. This directly implies that in those cases, energetic benefits of PP_i_ recycling are limited to the carbon ending up in the biomass without additional gains in catabolism. Therefore, to get a more accurate prediction of the amount of PP_i_ required or produced during biosynthesis and the possible energetic benefits of this, a stoichiometric analysis was performed. As is common for existing stoichiometric *C. thermocellum* models (26–28), the cellular composition of the Gram-positive bacterium Bacillus subtilis was used (Table 1 and Fig. 2) (29). Lumped reactions from precursor metabolites to the cell building blocks (i.e., amino acids, nucleotides, lipids, lipoteichoic acids, and cell wall components) were used, and for each precursor metabolite and cell building block a PP_i_ stoichiometry was determined (see Tables S1 to S8 in the supplemental material). Since the PEP-to-pyruvate conversion can occur through both the PP_i_-consuming Ppdk reaction and the PP_i_-neutral malate shunt, a degree of freedom, *x*, was implemented that can vary between 0 (only the malate shunt is used) and 1 (only Ppdk is used).

**TABLE 1:** Pyrophosphate stoichiometry of biomass components formed from cellobiose

Macromolecule^*d*^	Biomass composition^*a*^ (%, wt/wt)	PP_i_ stoichiometry (mmol per g cells)		
		Biosynthesis of component^*b*^	Polymerization of component	Total
Protein	52.85	−3.471 − 4.556*x*	+4.688	1.218 − 4.556*x*
DNA	2.60	+0.067	+0.080	+0.147
RNA	6.55	+0.103	+0.195	+0.298
Lipids	7.60	−0.779 − 1.636*x*		−0.779 − 1.636*x*
Lipoteichoic acids	3.04	0.073 − 0.136*x*		0.073 − 0.136*x*
Cell wall components	22.42	0.166 − 0.938*x*		0.166 − 0.938*x*
Ash fraction^*c*^	4.94			
**Total**	**100**	**−3.841** − **7.264*x***	**+4.963**	**1.122** − **7.266*x***

a Composition taken from Oh et al. (29) for Bacillus subtilis grown in aerobic glucose-limited chemostat cultivations at a dilution rate of 0.10 h^−1^.

b For macromolecules derived from pyruvate, acetyl-CoA, or α-ketoglutarate, a degree of freedom (*x*) is included. This parameter describes the flux distribution between the Ppdk reaction and the malate shunt. If *x* = 1, the PEP-to-pyruvate conversion solely goes through Ppdk. If *x* = 0, the PEP-to-pyruvate conversion solely goes through the malate shunt.

c The ash fraction equals the ion and metabolite fraction reported by Oh et al. (29).

d A breakdown of the PP_i_ stoichiometries of each macromolecule is shown in Tables S2 to S8.

The stoichiometric analysis showed that PP_i_ is formed in polymerization reactions (for protein, DNA, and RNA synthesis) and in numerous biosynthetic reactions where NTP is converted to NMP and PP_i_ (i.e., in arginine, asparagine, cysteine, histidine, methionine, and tryptophan synthesis but also in nucleotide, lipid, lipoteichoic acid, and cell wall synthesis). However, synthesis of many of the precursor metabolites needed to form these cell building blocks will cost PP_i_ (Table 1 and Fig. 2). The overall PP_i_ stoichiometry of biosynthesis indicated that biosynthesis would only have a net formation of PP_i_ if the Ppdk reaction accounts for ≤15% of the PEP-to-pyruvate flux. This implies that for the previously reported PEP-to-pyruvate flux distribution determined for wild-type *C. thermocellum* (i.e., 67% through Ppdk; 33% through the malate shunt [8]), biosynthesis would actually require a net input of PP_i_.

**FIG 3:**
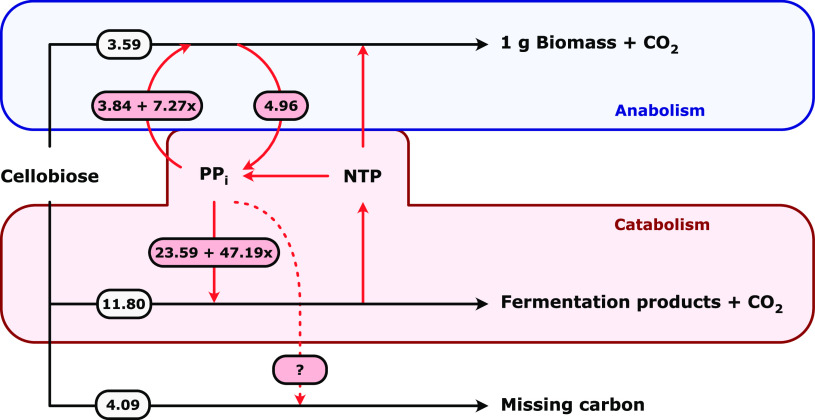
Schematic representation of the estimated pyrophosphate amounts (in mmol per g cells) needed in anabolism and catabolism for wild-type *C. thermocellum* grown in cellobiose-limited chemostat cultures (at a dilution rate of 0.1 h^−1^). The PP_i_ amounts depend on the parameter *x*, which describes the flux distribution between the Ppdk reaction and the malate shunt (*x* = 1, the PEP-to-pyruvate conversion solely goes through Ppdk; *x* = 0, the PEP-to-pyruvate conversion solely goes through the malate shunt). The amount of PP_i_ and cellobiose (in mmol) needed for anabolism is based on the stoichiometric analysis of biosynthesis (Fig. 2) using the macromolecular composition of aerobically grown glucose-limited chemostat cultures of B. subtilis (Table 1). The total amount of cellobiose (19.48 mmol) needed per gram of cells is based on an observed biomass yield of 0.15 g cells per g cellobiose obtained for cellobiose-limited chemostat cultures of wild-type *C. thermocellum* (30). The amount of cellobiose needed for catabolism (11.80 mmol) is calculated by subtracting the anabolic requirement (3.59 mmol) and unaccounted carbon (21% of the total, i.e., 4.09 mmol) from the total amount of cellobiose. Red arrows represent PP_i_ amounts in mmol g*_x_*^−1^; black arrows represent cellobiose amounts in mmol g*_x_*^−1^. Figure adapted from Holwerda et al. (18).

When the anabolic and catabolic PP_i_ requirements are combined (Fig. 3), for an experimentally determined biomass yield of 0.15 g cells per g cellobiose (30), it can be seen that even if the whole PEP-to-pyruvate flux passes through the malate shunt (i.e., *x* = 0), biosynthesis can only account for maximally 4.7% of the PP_i_ required in catabolism. Therefore, the updated PP_i_ stoichiometry of biosynthesis also implies that *C. thermocellum* requires a currently unknown (net) nonbiosynthetic PP_i_ source to supply the remaining catabolic PP_i_ needed to drive its glycolysis. This main conclusion is robust and independent of small changes in cellular composition resulting from, for instance, deviations from the commonly used B. subtilis composition or changes due to differing growth conditions.

### Prediction of putative PP_i_ sources using the optStoic procedure.

In addition to the previously suggested sources of nonbiosynthetic PP_i_, i.e., the H^+^-pumping membrane-bound PPase and glycogen cycling (9), which will be investigated in this work, we also searched for alternative PP_i_-supplying pathways. In selecting candidate pathways, the following considerations were taken into account. First, a possible PP_i_-supplying pathway must be able to carry enough flux to drive glycolysis. Second, PP_i_ needs to be generated directly or indirectly from ATP or GTP (9) with an overall stoichiometry of at least ${\text{ATP}}_{\text{eq}}+{\text{P}}_{\text{i}}\to {\text{ADP}}_{\text{eq}}+{\text{PP}}_{\text{i}}$ (or energetically more beneficial). This precludes, for instance, direct hydrolysis of an ATP to AMP and PP_i_ (${\text{ATP}}_{\text{eq}}\to {\text{AMP}}_{\text{eq}}+{\text{PP}}_{\text{i}}$), since, after balancing by adenylate kinase, this leads to the consumption of two ATP_eq_ per mol PP_i_ (13), thereby making this mechanism incompatible with a high ATP-yielding glycolysis (equation 2).

**FIG 4:**
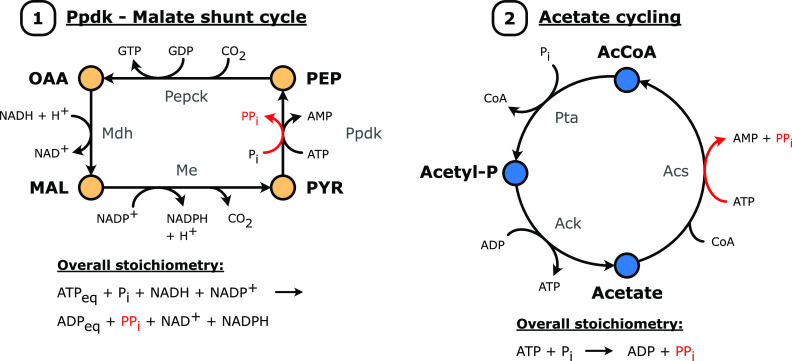
Two putative pyrophosphate-supplying pathways as identified with the help of the optStoic algorithm (31). (1) Ppdk–malate shunt cycle, where the malate shunt operates in the forward direction (PEP-to-pyruvate) and Ppdk in the reverse direction (pyruvate-to-PEP). (2) Acetate cycling, which occurs via the two-step conversion of acetyl-CoA to acetate, catalyzed by phosphotransacetylase (Pta) and acetate kinase (AcK), and the subsequent conversion of acetate to acetyl-CoA, catalyzed by acetyl-CoA synthetase (Acs). Both pathways result in formation of one PP_i_ from one ATP equivalent. For the Ppdk–malate shunt cycle, a simultaneous transhydrogenation of NADH and NADP^+^ to NAD^+^ and NADPH also occurs. ATP, ADP, and AMP were balanced in the overall stoichiometry with the adenylate kinase reaction (2 ADP ↔ ATP + AMP) and the nucleoside-diphosphate kinase reaction (ATP + NDP ↔ ADP + NTP). Abbreviations: AcCoA, acetyl-coenzyme A; Acetyl-P, acetyl-phosphate; CoA, coenzyme-A; MAL, malate; Mdh, malate dehydrogenase; Me, malic enzyme; OAA, oxaloacetate; PEP, phosphoenolpyruvate; Pepck, phosphoenolpyruvate carboxykinase; Ppdk, pyruvate phosphate dikinase; PYR, pyruvate.

To identify additional theoretical PP_i_-supplying pathways, we used the optStoic procedure (31) to search for the minimal set of reactions within the iCBI655 genome-scale metabolic model of *C. thermocellum* (26) that have an overall stoichiometry where one ATP and P_i_ is converted to one ADP and PP_i_. Of the top 100 proposed PP_i_-supplying mechanisms, 76 did not contain the membrane-bound PPase or glycogen cycling as key mechanisms (File S1). Of these 76 cycles, eight model solutions involved acetate cycling using acetyl-coenzyme A (CoA) synthetase as a key PP_i_-supplying enzyme (Fig. 4). Next, two malate shunt cycles were predicted, which convert pyruvate to PEP via Ppdk, PEP to oxaloacetate via PEP carboxykinase, and oxaloacetate to pyruvate via pyruvate carboxylase. Both cycles differ only in the enzymes used to balance the nucleotide cofactors. As pyruvate carboxylase activity has not been reported for *C. thermocellum*, we modified this cycle to include PEP carboxykinase, malate dehydrogenase, malic enzyme, and Ppdk (Fig. 4), which are known to be present and active in *C. thermocellum* (8). Two cycles required 3-isopropenyl-6-oxoheptanoate:CoA ligase, which is a fatty acyl-CoA ligase involved in limonene and pinene degradation. Given that *C. thermocellum* is not grown in the presence of these monoterpenes, it is highly unlikely that these cycles are active in *C. thermocellum*; therefore, they are not included for further study. Furthermore, 64 possible PP_i_-supplying cycles that require enzymes involved in nucleotide synthesis (i.e., NTP pyrophosphorylase and UTP pyrophosphohydrolase) were not investigated in the context of this study for two reasons. First, each PP_i_-supplying enzyme in these cycles is encoded by multiple gene candidates without an experimentally confirmed annotation. Identifying the correct gene candidate would be a time-consuming process given the current gene-editing techniques available for *C. thermocellum* (32). Second, their involvement in nucleotide biosynthesis might complicate interpretation of the physiological impact of these knockouts (if viable at all). Hence, four candidates were targeted for their role as a possible PP_i_ supplier: H^+^-pumping membrane-bound PPase, glycogen cycling, acetate cycling, and the Ppdk–Malate shunt cycle.

### Functional analysis of four possible individual sources of PP_i_ in *C. thermocellum*.

To investigate the contributions of the four proposed pathways (as described in the previous paragraph) in generating PP_i_, all genes predicted to encode the PP_i_-supplying enzymes in these pathways were individually deleted in wild-type strain LL1004, yielding strains AVM008 (Δ*clo1313_0823*; encoding putative membrane-bound pyrophosphatase), AVM051 (ΔP*_clo1313_0717-0718_*-*clo1313_0717-0718*; encoding putative ADP-glucose synthase, which consists of two subunits), AVM003 (Δ*clo1313_0949*; Δ*ppdk*), and AVM059 (Δ*clo1313_1686*; encoding putative acetyl-CoA synthetase).

To verify that the introduced genetic modifications resulted in a complete loss of the predicted activity and simultaneously functionally annotate the targeted genes, enzyme activities were measured in cell extracts of batch serum bottle cultures (Table 2). In line with previous reports (8), deletion of *clo1313_0949*, encoding Ppdk, completely removed the Ppdk activity in AVM003 compared to LL1004 (Table 2). Furthermore, removal of *clo1313_0823* resulted in a complete elimination of pyrophosphatase activity in AVM008 compared to LL1004 (Table 2), providing *in vitro* evidence that *clo1313_0823* encodes a functional pyrophosphatase. Despite numerous assay optimization attempts (see Materials and Methods), ADP-glucose synthase activity could not be measured in cell extracts of the wild-type strain LL1004 (data not shown).

**TABLE 2:** PPase and Ppdk activities of cell extracts from *C. thermocellum* wild-type and mutant strains^*a*^

Strain	Relevant genotype	Enzyme activity (μmol mg protein^−1^ min^−1^)	
		PPase	Ppdk
LL1004	Wild-type	0.037 ± 0.007	0.40 ± 0.04
AVM008	Δ*ppase* (Δ*clo1313_0823*)	<0.005	ND^*b*^
AVM003	Δ*ppdk* (Δ*clo1313_0949*)	ND	<0.05
AVM061	Δ*ppase* Δ*P_ags1_*_,_*_2_-ags1 ags2* Δ*ppdk* Δ*clo1313_1686*	<0.005	<0.05

a Averages and standard deviations were obtained from two independent biological duplicates. The detection limit was 0.05 μmol mg protein^−1^ min^−1^ for the Ppdk assay and 0.005 μmol mg protein^−1^ min^−1^ for the PPase assay.

b ND, not determined.

**FIG 5:**
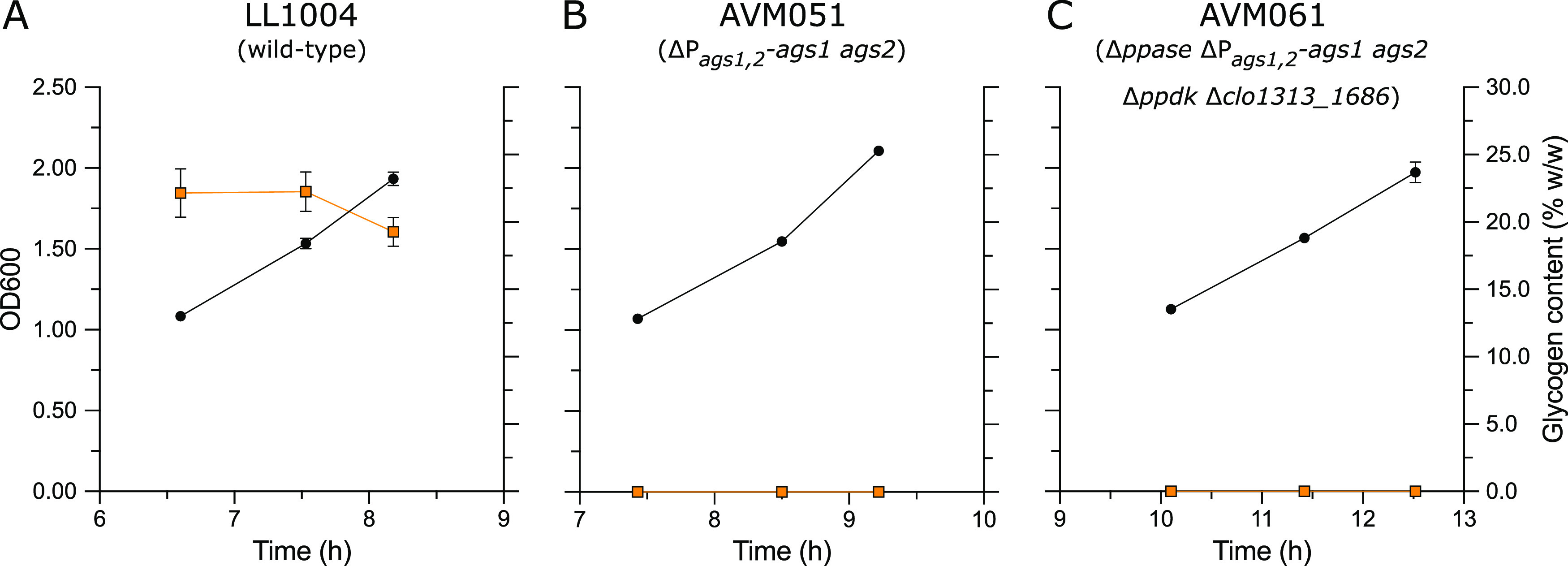
Growth (OD_600_) and glycogen formation of LL1004 (wild type) (A), AVM051 (Δ*P_ags1_*_,_*_2_-ags1 ags2*) (B), and AVM061 (Δ*ppase* Δ*P_ags1_*_,_*_2_-ags1 ags2* Δ*ppdk* Δ*clo1313_1686*) (C). Cultures were grown on LC medium containing 5 g L^−1^ cellobiose. Symbols: black circles, OD_600_; orange squares, glycogen content. Data are shown for one representative experiment (*n *= 3). Averages and standard deviations for each data point were obtained from technical triplicate measurements.

As an alternative to enzymatic activity measurements of ADP-glucose synthase, glycogen formation was measured during exponential growth of LL1004 and AVM051 (ΔP*_clo1313_0717-0718_*-*clo1313_0717-0718*). In contrast to the wild-type strain LL1004, which formed 20% to 25% (wt/wt) glycogen during the exponential growth phase, biomass of the ADP-glucose synthase deletion strain (AVM051) did not contain glycogen (Fig. 5). Since ADP-glucose synthase activity is essential for a functioning glycogen cycle, this is a strong, albeit indirect, indication that removal of P*_clo1313_0717-0718_*-*clo1313_0717-0718* eliminated ADP-glucose synthase activity. For functional analysis of *clo1313_0717* and *clo1313_0718*, encoding the two subunits of the *C. thermocellum* ADP-glucose synthase, both genes were simultaneously expressed from the high-copy-number pTrc99a plasmid in E. coli BL21. ADP-glucose synthase activity was readily detected in E. coli cell extracts expressing *clo1313_0717* and *clo1313_0718*, whereas this activity was not detected in the empty vector control when assayed at 55°C (Table S9). Furthermore, the activity was found to be ADP-glucose dependent, as GDP- and UDP-glucose could not be used as the substrate. This indicated that the *C. thermocellum* genes *clo1313_0717* and *clo1313_0718* indeed encode an ADP-glucose synthase.

**FIG 6:**
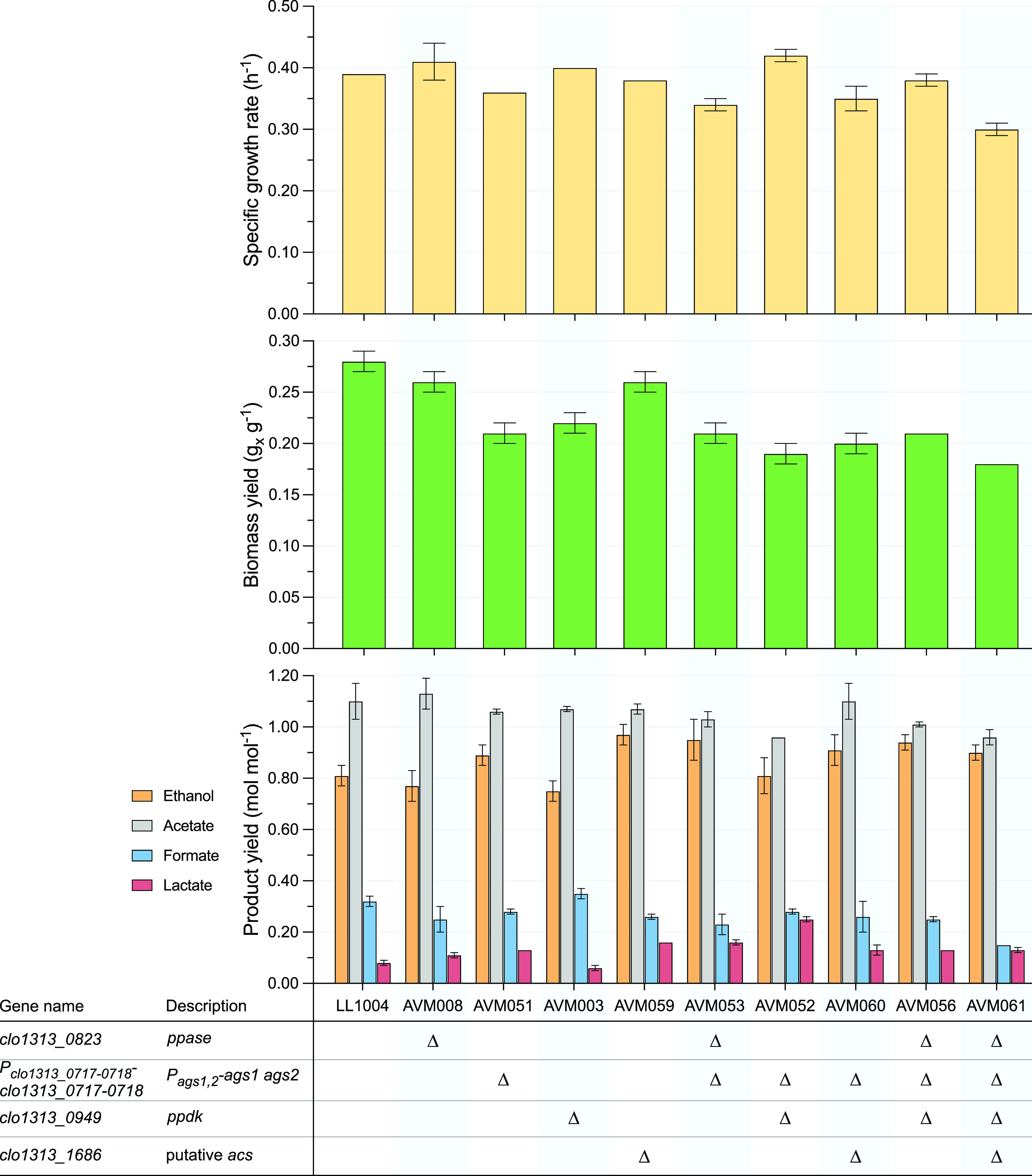
Maximum specific growth rates, biomass yields, and fermentation product yields of *C. thermocellum* wild-type and mutant strains in batch serum bottle cultures. Cultures were grown on LC medium containing 5 g L^−1^ cellobiose. Averages and standard deviations were obtained from three independent biological replicates. Absence of error bars indicates standard deviations were equal to zero.

For a quantitative analysis of the impact of these three individual gene deletions, the specific growth rate, biomass yield, and fermentation product yields were determined in batch serum bottle cultures (Fig. 6 and Table S10). Interestingly, single knockouts of the membrane-bound PPase (AVM008), ADP-glucose synthase (AVM051), and Ppdk (AVM003) did not have a major effect on the observed growth rates compared to LL1004 (Fig. 6). If one of these sources was indeed solely responsible for generating PP_i_, one would expect a lower growth rate in the knockout strains resulting from a decreased PP_i_ flux and a consequently lower glycolytic flux, which was not observed for these mutants. Consistent with previous reports (18), the biomass yield in AVM008 (Δ*ppase*) was similar to that of the wild-type strain (LL1004) (Fig. 6). Given that PP_i_ supply via the membrane-bound PPase was hypothesized to have a PP_i_-to-ATP ratio higher than unity (2), we expected a decreased biomass yield in AVM008. The absence of this effect suggested that there is no or very low contribution of this mechanism to PP_i_ supply in *C. thermocellum*. Knockouts of ADP-glucose synthase (AVM051) and Ppdk (AVM003) resulted in a 24% (*P < *0.01) and 20% (*P < *0.01) decrease, respectively, in biomass yield compared to LL1004 (Fig. 6). Considering that glycogen as a biomass component is energetically inexpensive to make compared to other cell building blocks (e.g., protein, DNA, RNA, etc.), removal of glycogen in AVM051 (ΔP*_clo1313_0717-0718_*-*clo1313_0717-0718*; ΔP*_ags1_*_,_*_2_-ags1 ags2*) was expected to make formation of new cells without glycogen more energetically costly, leading to a lower biomass yield. The slightly reduced biomass yield in AVM003 (Δ*ppdk*) was consistent with earlier reports (8). Finally, no major shifts in the measured fermentation product yields were observed for the three single knockout strains compared to LL1004 (Fig. 6 and Table S10). These results indicate that neither the H^+^-pumping membrane-bound PPase, glycogen cycling, nor the Ppdk–Malate shunt cycle is essential for PP_i_ generation.

The fourth possible mechanism, acetate cycling, with acetyl-CoA synthetase (Acs) as the key enzyme (Fig. 4), was also investigated. As commonly used Acs assays measure combined activity of acetate kinase (Ack) and phosphotransacetylase (Pta) (33), acetyl-CoA synthetase activity was first measured in a *pta* knockout background (strain LL1041 [34]) (Table S11). Interestingly, no measurable acetyl-CoA synthetase activity was detected in these cell extracts. In agreement with this result, no difference in Acs-like enzyme activity was observed between strains LL1004 (wild type) and AVM059 (Δ*clo1313_1686*), indicating that the measured activity for these strains was due to Pta-Ack activity (Table S11). The absence of measurable Acs activity likely cannot be ascribed to low gene expression or translation, as *clo1313_1686* is highly expressed at the transcriptional level (6, 35) and moderately expressed at the translational level (36). For further functional analysis, *clo1313_1686* was expressed in E. coli BL21 from the pTrc99a plasmid with parallel expression of the native E. coli *acs* gene (*b21_03901*) as a control. SDS-PAGE analysis showed that *clo1313_1686* was highly expressed in the BL21 pTK54 cell extract (Fig. S1). However, at 55°C, no Acs activity was observed for any of the E. coli cell extracts (Table S12). In contrast to the 3-fold higher Acs activity at 37°C when overexpressing the native E. coli *acs* gene (BL21 pTK55), activity at 37°C in E. coli expressing *clo1313_1686* (BL21 pTK54) was similar to that of the empty vector control (BL21 pTrc99a) (Table S12). Based on these results, *clo1313_1686* likely does not encode an acetyl-CoA synthetase, and the lack of Acs activity makes acetate cycling as a PP_i_-supplying mechanism unlikely. In line with this, deletion of *clo1313_1686* (AVM059) in *C. thermocellum* did not have a significant effect on the growth rate, biomass yield, or fermentation product yields compared to LL1004 (Fig. 6 and Table S10). However, to fully exclude any possibility that this gene contributes to a PP_i_-supplying mechanism, the *clo1313_1686* gene knockout was still included in subsequent combinatorial deletion studies.

### Physiological impact of the combinatorial deletion of four possible PP_i_ sources in *C. thermocellum*.

A possible explanation for the observed lack of a phenotypic effect upon disrupting the single putative PP_i_-supplying pathways is that they can functionally complement each other. To test this hypothesis, double, triple, and quadruple knockout strains were created, resulting in strains AVM053 (Δ*ppase* Δ*P_ags1_*_,_*_2_-ags1 ags2*), AVM052 (Δ*ppdk* Δ*P_ags1_*_,_*_2_-ags1 ags2*), AVM060 (Δ*P_ags1_*_,_*_2_-ags1 ags2* Δ*clo1313_1686*), AVM056 (Δ*ppase* Δ*P_ags1_*_,_*_2_-ags1 ags2* Δ*ppdk*), and AVM061 (Δ*ppase* Δ*P_ags1_*_,_*_2_-ags1 ags2* Δ*ppdk* Δ*clo1313_1686*). Double knockouts of possible PP_i_-supplying pathways had no large detrimental effects on the growth rates, biomass yields, or fermentation product yields (Fig. 6 and Table S10). For the double knockouts AVM053 (Δ*ppase* Δ*P_ags1_*_,_*_2_-ags1 ags2*) and AVM052 (Δ*ppdk* Δ*P_ags1_*_,_*_2_-ags1 ags2*), removal of ADP-glucose synthase lowered the biomass yield, with 20% (*P < *0.01) and 15% (*P < *0.01) compared to the single-knockout strains AVM008 (Δ*ppase*) and AVM003 (Δ*ppdk*), respectively. This decrease was in line with the decreased biomass yield observed for the single deletion of ADP-glucose synthase (AVM051). Interestingly, deletion of *ppdk* in AVM053 (Δ*ppase* Δ*P_ags1_*_,_*_2_-ags1 ags2*), creating the triple-knockout strain AVM056, also did not result in a major phenotypic change (Fig. 6 and Table S10). Finally, deletion of *clo1313_1686* in the triple-knockout strain AVM056 (Δ*ppase* Δ*P_ags1_*_,_*_2_-ags1 ags2* Δ*ppdk*), resulting in AVM061, did lower the growth rate to 0.30 h^−1^ and the biomass yield to 0.18 g*_x_* g^−1^, which was ca. 22% (*P < *0.01) and 15% (*P < *0.01) lower than that of AVM056. Furthermore, fermentation product yields were comparable between these strains (Fig. 6 and Table S10). The lower growth rate and biomass yield observed upon deletion of *clo1313_1686* in AVM056 are in contrast to the effect of this knockout observed in the wild type (LL1004) and AVM051 (Δ*P_ags1_*_,_*_2_-ags1 ags2*) (Fig. 6). In agreement with the single-knockout strains, no enzyme activity of Ppdk and PPase or formation of glycogen was observed in AVM061 (Δ*ppase* Δ*P_ags1_*_,_*_2_-ags1 ags2* Δ*ppdk* Δ*clo1313_1686*) (Fig. 5, Table 2). Additionally, the combined Acs/Pta-Ack activity in strains AVM056 (Δ*ppase* Δ*P_ags1_*_,_*_2_-ags1 ags2* Δ*ppdk*) and AVM061 (Δ*ppase* Δ*P_ags1_*_,_*_2_-ags1 ags2* Δ*ppdk* Δ*clo1313_1686*) was comparable to that of strains LL1004 and AVM059 (Δ*clo1313_1686*) (Table S11), which further confirms that it is unlikely that, in a strain with the other three mechanisms deleted, acetate cycling contributes to PP_i_ supply. Compared to the wild-type strain LL1004, the quadruple deletion strain AVM061 (Δ*ppase* Δ*P_ags1_*_,_*_2_-ags1 ags2* Δ*ppdk* Δ*clo1313_1686*) showed a 22% (*P < *0.01) and 38% (*P < *0.01) decreased growth rate and biomass yield. The ethanol yield only slightly increased from 0.81 to 0.90 mol mol^−1^ (*P < *0.05), and the acetate yield decreased from 1.10 to 0.96 mol mol^−1^ (*P < *0.05) relative to LL1004 (Fig. 6). The small, combined effects of the four deletions in AVM061 were much smaller than what would be expected if one or more of these genes play a role in supply of the PP_i_ needed to drive a PP_i_-dependent glycolysis.

### Continued cellobiose fermentation in the quadruple knockout strain during growth arrest.

Although our metabolic network analysis showed that biosynthesis cannot supply enough PP_i_ to drive glycolysis (Fig. 2 and 3 and Table 1), it might be that current knowledge and/or the assumptions used for the biosynthetic pathways are not correct. If biosynthesis can in fact supply enough PP_i_, which would explain the lack of a severe phenotypic effect in the quadruple knockout strain AVM061 (Δ*ppase* Δ*P_ags1_*_,_*_2_-ags1 ags2* Δ*ppdk* Δ*clo1313_1686*) compared to LL1004, one would expect that fermentation of cellobiose cannot occur in growth-arrested cells of AVM061.

To test this hypothesis, first cells of the wild-type strain (LL1004) were harvested, washed, and transferred to (i) unmodified LC medium, (ii) LC medium without urea as the sole nitrogen source, and (iii) LC medium without urea and cellobiose (sole carbon source). Similar to previous studies (9), we observed nitrogen depletion to be an effective method to arrest growth with continued fermentation (Fig. S2). However, after 24 h, fermentation significantly slowed down and even stopped, while about half of the initial cellobiose remained. As key glycolytic enzymes are regulated by intracellular NH_4_^+^ levels (37, 38), we hypothesized that this could be a confounding effect of the use of nitrogen depletion to control cell growth. Therefore, we considered other nutrients such as sulfur to achieve growth arrest.

**FIG 7:**
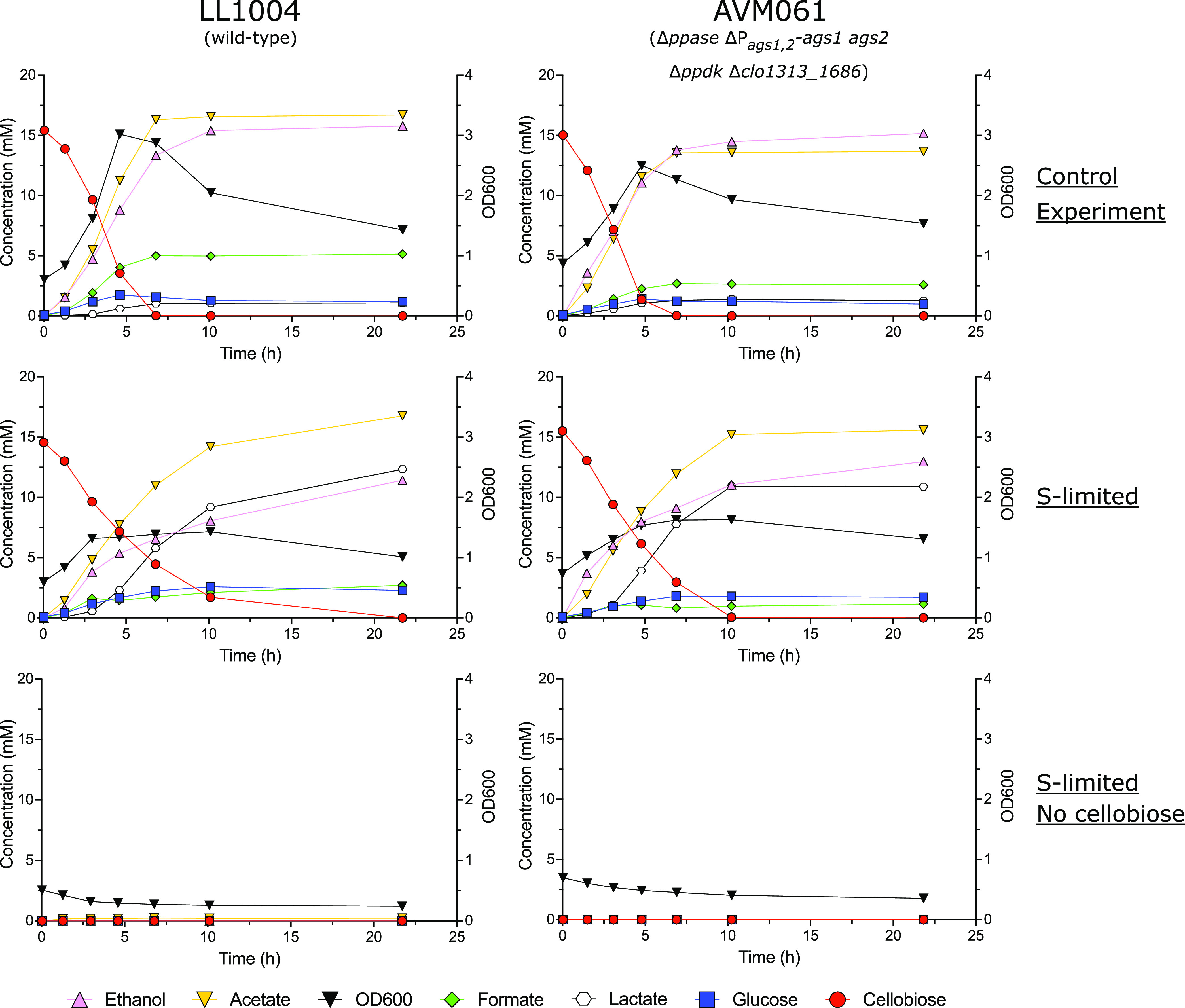
Growth and product profiles of LL1004 (wild type; left column) and AVM061 (Δ*ppase* Δ*P_ags1_*_,_*_2_-ags1 ags2* Δ*ppdk* Δ*clo1313_1686*; right column) during growth arrest studies. Cultures were grown on LC medium (top; control experiment), LC medium without Na_2_SO_4_ and with 0.01 g L^−1^ cysteine (middle; S-limited), and LC medium without cellobiose and Na_2_SO_4_ but with 0.01 g L^−1^ cysteine (bottom; S-limited, no cellobiose). Data are shown for one representative experiment (*n *= 2).

The main sulfur sources in LC medium are Na_2_SO_4_ and cysteine (39). Since cysteine is also used as a reducing agent, it was not completely removed, but instead cysteine levels were decreased 10-fold and Na_2_SO_4_ was completely removed. For this method, cells of LL1004 and AVM061 (Δ*ppase* Δ*P_ags1_*_,_*_2_-ags1 ags2* Δ*ppdk* Δ*clo1313_1686*) were harvested, washed, and transferred to three different LC media: (i) unmodified LC medium, (ii) LC medium without Na_2_SO_4_ and with only 0.01 g L^−1^ cysteine, and (iii) LC medium without cellobiose and Na_2_SO_4_ but with 0.01 g L^−1^ cysteine. When transferred to unmodified LC medium, strains LL1004 and AVM061 both grew exponentially from the start, increasing the OD_600_ from 0.61 to 3.02 and 0.88 to 2.50, respectively, and rapidly consuming all cellobiose (Fig. 7). Both strains produced roughly equimolar amounts of acetate (16.7 mM and 13.7 mM for LL1004 and AVM061, respectively) and ethanol (15.8 mM and 15.2 mM for LL1004 and AVM061, respectively) as main fermentation products. Transfer to sulfur-limited LC medium initially resulted in an OD_600_ increase from 0.60 to 1.32 and 0.74 to 1.54 for LL1004 and AVM061, respectively (Fig. 7). In this initial period, the remaining sulfur sources (mainly cysteine and trace amounts from metal sulfates) were completely consumed. After this initial period, growth was arrested for both strains and cells continued to ferment cellobiose to acetate, ethanol, and lactate as main fermentation products. Lastly, cells transferred to the control medium without cellobiose and with limited sulfur sources produced only minor amounts of acetate (0.24 mM) in LL1004 and did not produce any fermentation products in AVM061 (Fig. 7). The tiny amount of acetate in LL1004 was likely the result of conversion of intracellularly stored glycogen in the inoculum. Overall, these results show that for both the wild-type strain (LL1004) and the quadruple knockout strain (AVM061), cellobiose fermentation can still occur in growth-arrested cells, which excludes biosynthesis as a primary source of PP_i_ for glycolysis.

### Analysis of phosphofructokinase activity in *C. thermocellum*.

Previous studies on organisms with PP_i_-dependent glycolysis have shown that in addition to a PP_i_-dependent phosphofructokinase (Pfk), some of these organisms also possess a functional ATP- or GTP-dependent 6-phosphofructokinase (40–43). The presence of a functional ATP- or GTP-dependent Pfk could decrease the PP_i_ demand in glycolysis and thereby provide an explanation for the observed phenotype of AVM061 (Δ*ppase* Δ*P_ags1_*_,_*_2_-ags1 ags2* Δ*ppdk* Δ*clo1313_1686*). Although ATP-dependent Pfk activity has hitherto never been measured in *C. thermocellum*, one gene has been annotated as an ATP-dependent Pfk in the KEGG (44, 45) and Pfam databases (46, 47), *clo1313_0997*, and this gene is moderately expressed at the transcriptional and translational level (6, 35, 36). Furthermore, two other candidate genes, *clo1313_1832* and *clo1313_2627*, are both annotated as members of the carbohydrate kinase PfkB family (48) in the Pfam database, whereas the KEGG database annotated these genes as a fructokinase and tagatose-6-phosphate kinase, respectively. Both genes are expressed at low to moderate levels (6, 35, 36).

To investigate if ATP- or GTP-dependent Pfk activity contributes to the observed phenotypes, cell extracts of strains LL1004 and AVM061 (Δ*ppase* Δ*P_ags1_*_,_*_2_-ags1 ags2* Δ*ppdk* Δ*clo1313_1686*) were assayed for PP_i_-, ATP-, and GTP-dependent Pfk activity. Pfk activity was found to be PP_i_ dependent only for both strains (Table 3). In line with this observation, heterologous expression of *clo1313_0997*, *clo1313_1832*, and *clo1313_2627* from the pTrc99a plasmid in E. coli BL21 showed similar ATP- and GTP-dependent Pfk activities as the empty vector control (Table S13). An additional protein denaturation heating step, aimed to remove the native E. coli background activity (49), also did not result in measurable Pfk activity in strains expressing the three *C. thermocellum* genes (Table S13). Heterologous expression in E. coli of the positive-control *tsac_1362*, encoding the *T. saccharolyticum* ATP- and GTP-dependent Pfk, showed a 5-fold higher ATP-dependent activity and an 11-fold higher GTP-dependent activity than the empty vector control (Table S13). This difference in activity became even more pronounced with the heat-treated cell extracts (Table S13). SDS-PAGE analysis showed the presence of all heterologous proteins in the E. coli cell extracts, albeit at varying protein levels (Fig. S3 and S4). Together, these measurements imply that neither *clo1313_0997*, *clo1313_1832*, nor *clo1313_2627* encodes an ATP- or GTP-dependent Pfk. Finally, ATP- as well as GTP-dependent fructokinase activity of 1.5 μmol mg protein^−1 ^min^−1^ was measured in E. coli expressing *clo1313_1832*, whereas it was below the detection limit of activity in the wild-type strain (LL1004), which provides biochemical confirmation of the current annotation in the KEGG database of *clo1313_1832* as a fructokinase (44, 45) (Table S14).

**TABLE 3:** PP_i_-, ATP-, and GTP-dependent phosphofructokinase activities of cell extracts from *C. thermocellum* LL1004 and AVM061^*a*^

Strain	Relevant genotype	Enzyme activity (μmol mg protein^−1^ min^−1^)		
		PP_i_	ATP	GTP
LL1004	Wild-type	3.55 ± 0.77	<0.05	<0.05
AVM061	Δ*ppase* Δ*P_ags1_*_,_*_2_-ags1 ags2* Δ*ppdk* Δ*clo1313_1686*	2.55 ± 0.22	<0.05	<0.05

a Averages and standard deviations were obtained from two independent biological duplicates. The detection limit was 0.05 μmol mg protein^−1 ^min^−1^.

### Whole-genome sequencing of the engineered strains.

The genome of all engineered strains used in this study was sequenced to verify the intended deletions and to identify secondary mutations acquired during strain construction (File S2). None of the observed secondary mutations were related to possible PP_i_-supplying pathways or to a lower PP_i_ demand (e.g., ATP/GTP-Pfk candidate genes, *clo1313_0997* and *clo1313_2627*, and their up- and downstream regions did not contain mutations). All engineered strains had acquired a nucleotide substitution in the *clo1313_1795* gene, encoding the hydrogenase-Fe-S B (hfsB) protein, which resulted in an I516K point mutation. Interestingly, this mutation was already present in the parental strain LL1004, albeit at low frequency (12%). Previous studies reported that disruption of *hfsB* in *C. thermocellum* resulted in increased ethanol production (6, 50). However, it is unlikely that the I516K point mutation had a similar effect, as the ethanol yields for the engineered strains are comparable to LL1004 (Fig. 6). Furthermore, AVM052 (Δ*ppdk* Δ*P_ags1_*_,_*_2_-ags1 ags2*) and the parental strain AVM003 (Δ*ppdk*) contained over three times more secondary mutations than LL1004. The increased mutation frequency of both strains is likely related to a single-nucleotide deletion in *clo1313_1445*, a gene that encodes the DNA mismatch repair protein MutS. This single-nucleotide deletion resulted in a frameshift of the gene that could inactivate the protein. Mutations in the *mutS* gene have been shown to result in failure to repair replication errors and give rise to strong mutator or hypermutator phenotypes (51, 52).

### Prediction of additional PP_i_-generating cycles using an adapted optStoic procedure.

optStoic results are inherently dependent upon the quality of the genome annotation. Given that bioinformatically predicted annotations are not always correct, as seen for *clo1313_1686*, and that approximately 20% of the *C. thermocellum* genome encodes proteins of unknown function (also called hypothetical proteins) (53), it is possible that important PP_i_-supplying mechanisms are currently missed. In an attempt to address this possibility, the KEGG database was probed for PP_i_-generating cycles carrying out the net conversion, ${\text{ATP}}_{\text{eq}}+{\text{P}}_{\text{i}}\to {\text{ADP}}_{\text{eq}}+{\text{PP}}_{\text{i}}$, which would be feasible in *C. thermocellum* (26) with the addition of one reaction currently absent from iCBI655. Of the 78 proposed cycles (File S3), 66 can be eliminated, as they required the use of the membrane-bound PPase or ADP-glucose synthase, which have been deleted in this study. One cycle required sulfate adenylyltransferase, which, due to its essential function in sulfur metabolism, cannot be deleted. The 11 remaining PP_i_-supplying cycles are involved in nucleotide synthesis or mannose metabolism and have multiple gene candidates encoding key enzymes. Hence, candidates identified with the addition of one reaction currently absent in iCBI655 have the same experimental limitations as those discussed for other candidate cycles not investigated in the context of this study.

## DISCUSSION

Four possible PP_i_-generating mechanisms were investigated: the H^+^-pumping membrane-bound PPase, glycogen cycling, a predicted Ppdk–malate shunt cycle, and acetate cycling with acetyl-CoA synthetase as a key enzyme. Several convergent lines of evidence indicate that none of these are the major source of PP_i_ in *C. thermocellum*. Knockout of a significant PP_i_-supplying mechanism was expected to severely impair growth or lead to no growth. However, the observed 22% and 38% decreased growth rate and biomass yield in AVM061 (Δ*ppase* ΔP*_ags1_*_,_*_2_-ags1 ags2* Δ*ppdk* Δ*clo1313_1686*) compared to the wild type (LL1004) show that the targeted sources are not important for PP_i_ supply and that there are still one or multiple PP_i_-supplying pathways functional in *C. thermocellum*. The lower biomass yield and growth rate in AVM061 are, in addition to the already-described decrease resulting from glycogen removal, likely linked to decreased metabolic flexibility or possible unknown side effects of the deletions.

The 20% decreased biomass yield observed upon deletion of *ppdk* in LL1004, which was also observed by Olson et al. (8), is one example of the impact of decreased metabolic flexibility but might simultaneously provide information about the stoichiometry of an unknown PP_i_-generating mechanism(s) (Fig. 6). The malate shunt, which, in a *ppdk* knockout strain, carries the entire PEP-to-pyruvate flux, yields one ATP equivalent and transhydrogenation of all glycolytically formed NADH to NADPH (Fig. 1). In contrast, the Ppdk reaction converts PEP and PP_i_ to net formation of two ATP equivalents (with adenylate kinase balancing ATP, ADP, and AMP). The observed biomass yield decrease can be related to a catabolic oversupply of NADPH in Δ*ppdk* strains due to the different overall stoichiometry of the malate shunt. The excess NADPH can be reoxidized by producing amino acids (18) or by using NfnAB, thereby forming reduced ferredoxin and NADH, which likely leads to more hydrogen formation (54). Although neither product is measured in this study, increased amino acid and H_2_ formation were indeed observed previously in a *ppdk* deletion strain (8). The 9% lower carbon recovery in strain AVM003 (Δ*ppdk*) compared to strain LL1004 (Table S10) would be in line with such an increased amino acid excretion. Interestingly, if a currently unknown source of PP_i_ results in formation of more than 1 PP_i_ per ATP (or does not consume ATP), one would also expect a decreasing biomass yield upon deletion of *ppdk*, thereby providing an alternative (or additional) hypothesis for this observation.

In addition to the functional annotation of the membrane-bound PPase, ADP-glucose synthase, and a fructokinase, it was shown that *clo1313_1686* does not encode acetyl-CoA synthetase activity, which contradicts the current annotation in the KEGG database (45). A lack of measurable acetyl-CoA synthetase activity makes acetate cycling as a PP_i_-supplying mechanism unlikely. Interestingly, in contrast to the absence of an effect observed upon deleting *clo1313_1686* in the wild type (LL1004) and strain AVM051 (Δ*P_ags1_*_,_*_2_-ags1 ags2*), knockout of this gene in strain AVM056 (Δ*ppase* ΔP*_ags1_*_,_*_2_-ags1 ags2* Δ*ppdk*) lowered the growth rate and biomass yield. Since a functional annotation of this gene is currently missing, it is difficult to understand the observed effect of this knockout. Alternatively, whole-genome sequencing showed some secondary mutations that were only present in AVM061 (Δ*ppase* ΔP*_ags1_*_,_*_2_-ags1 ags2* Δ*ppdk* Δ*clo1313_1686*), which might explain the lower growth rate and biomass yield; however, none of these secondary mutations could be directly linked to this phenotype.

A currently unknown alternative PP_i_-supplying mechanism will have to meet two requirements. The first is that the overall stoichiometry of this mechanism will likely be equivalent to $\text{ATP}+{\text{P}}_{\text{i}}\to \text{ADP}+{\text{PP}}_{\text{i}}$. Stoichiometric ATP-to-PP_i_ ratios smaller than unity would contradict the observed reversibility and predicted high energetic efficiency of PP_i_-dependent glycolysis (11, 55). Furthermore, no other known alternative mechanism apart from the studied H^+^-pumping membrane-bound PPase would allow a stoichiometric ATP-to-PP_i_ ratio higher than unity. The second requirement is that the PP_i_-supplying mechanism must be able to carry sufficient flux to be able to drive glycolysis. For AVM061 (Δ*ppase* ΔP*_ags1_*_,_*_2_-ags1 ags2* Δ*ppdk* Δ*clo1313_1686*), one can estimate this flux to be approximately 9.7 mmol PP_i_ g*_x_*^−1^ h^−1^ based on an observed growth rate of 0.30 h^−1^, a biomass yield of 0.18 g*_x_* g_s_^−1^, and the assumption that PP_i_-Pfk is the only PP_i_-consuming reaction in the absence of Ppdk. PP_i_-dependent acetate kinase (EC 2.7.2.12) and a PP_i_-dependent PEP carboxykinase (PEPCK) (EC 4.1.1.38) have previously been suggested to supply PP_i_ in Entamoeba histolytica (56, 57) and *Propionibacterium shermanii* (58). However, in *C. thermocellum* only one gene is annotated as acetate kinase and was found to be ATP dependent (10, 59), and PEPCK was unable to use orthophosphate as the substrate (9), which likely eliminates these possibilities. The nonoxidative pentose phosphate pathway of *C. thermocellum* has previously been shown to use PP_i_-PFK instead of transaldolase (60), thereby contributing to the PP_i_ originating from biosynthesis (61). However, the optStoic analysis did not identify a possible cyclic pathway involving these reactions as a potential additional PP_i_ source. Since all remaining qualifying PP_i_-supplying options identified using optStoic with at most a single reaction absent from the iCBI655 model are either part of biosynthetic networks, have essential functions, or rely on unavailable precursor molecules, their future investigation will need further gene-editing developments or alternative scientific approaches.

In contrast to having an alternative PP_i_-generating mechanism, a smaller than foreseen glycolytic PP_i_ demand is also possible by having an active ATP/GTP-dependent Pfk. Although this activity was not found in this study (Table 3; see also Table S13 in the supplemental material), it is not uncommon for bacteria and eukaryotes with a PP_i_-dependent glycolysis to have at least one gene encoding an ATP/GTP-dependent Pfk. When cell extracts of these organisms were assayed for ATP- and PP_i_-dependent Pfk activity, either no ATP-dependent activity was found (9, 49, 62) or this activity was 6- to 11-fold lower than that of the PP_i_-dependent activity (42, 63). For Clostridium thermosuccinogenes, ATP-Pfk activity was only detected after protein purification; however, the catalytic efficiency (defined as *k*_cat_/*K_m_*) of ATP-Pfk was much lower than that of purified PP_i_-Pfk (60). Additionally, ATP-Pfk activity of the purified E. histolytica ATP-Pfk was only detected after an initial preincubation step with ATP and at relatively high fructose-6-phosphate concentration (20 mM) (42). Hence, it could be that biochemical knowledge and methodologies are currently missing, preventing the measurement of ATP/GTP-Pfk activity in *C. thermocellum*.

Although the H^+^-pumping membrane-bound PPase and ADP-glucose synthase do not have a PP_i_-supplying role, the fact that the genes encoding these enzymes are highly expressed and translated (35, 36) suggests other functions in *C. thermocellum*. Given that *C. thermocellum* has a PP_i_-dependent Pfk as the main PP_i_ sink, it is unlikely that the membrane-bound PPase functions solely as a PP_i_ disposal system. Baykov et al. (64) hypothesized that membrane-bound PPases play an important role in cell survival under stress conditions by utilizing the energy released upon PP_i_ hydrolysis to maintain ion gradients. Hence, it could be that the membrane-bound PPase in *C. thermocellum* is important for its robustness and that phenotypic effects of a *ppase* deletion strain are only observed under stress conditions. ADP-glucose synthase is important for glycogen formation in wild-type *C. thermocellum* (Fig. 5). Even though high-flux glycogen cycling is not an essential PP_i_-generating cycle, 20% to 25% (wt/wt) glycogen is formed in *C. thermocellum* during exponential growth (Fig. 5) and can be used as carbon and energy storage (65). As part of glycogen formation, ADP-glucose synthase does contribute to the anabolically produced PP_i_. For an average chain length of 10 glucose units (66) and a PP_i_ stoichiometry of 1 mol PP_i_ per glucose unit added to the glycogen chain, formation of 25% (wt/wt) glycogen can generate 0.15 mmol PP_i_ per g biomass, which would provide less than 1% of the total glycolytic PP_i_ requirement (Fig. 3).

The observation that the membrane-bound PPase is not responsible for PP_i_ supply in *C. thermocellum* brings into question the hypothesis that PP_i_-dependent glycolysis allows for a significantly higher energetic efficiency at the costs of thermodynamic driving force compared to an ATP-dependent glycolysis (11, 55, 67). Since membrane-bound PPase is currently the only predicted mechanism yielding a PP_i_-to-ATP ratio higher than unity, formation of PP_i_ via an alternative mechanism will likely make use of PP_i_ stoichiometrically equivalent to ATP. Hence, the only energetic benefit of using PP_i_ will come from the relatively small amount of biosynthetic PP_i_ that is recycled in glycolysis. However, *C. thermocellum* also has other mechanisms to conserve energy. One of these mechanisms is the phosphoroclastic cleavage of cellodextrins (as discussed in the introduction), which, depending on the oligomer size (*n*), could save 0.5 to 0.83 ATP per glucose equivalent (for *n *= 2 to 6) (68). Furthermore, coupling of the exergonic oxidation of reduced ferredoxin to endergonic reactions, e.g., ion translocation via membrane-bound hydrogenases (Ech) and oxidoreductases (Rnf) or transhydrogenation of NADH to NADPH (NfnAB) (54, 69), also allows for energy conservation. Therefore, although the energetic benefit of using PP_i_ might be small, it is likely one of many mechanisms that *C. thermocellum* has to optimize energy conservation from sugar dissimilation.

The present study has demonstrated that previously hypothesized PP_i_ sources are not responsible for PP_i_ supply in *C. thermocellum*. Together with the updated PP_i_ stoichiometry for biosynthesis, these findings can help to improve current genome-scale metabolic models as well as provide fundamental knowledge of the PP_i_ metabolism of *C. thermocellum*. optStoic-identified cycles with more than one reaction missing from the iCBI655 model can also serve as a starting point for performing sequence alignment to identify previously unknown reactions active in *C. thermocellum* and capable of cycling carbon to produce PP_i_. Furthermore, although our findings did not identify the source(s) of PP_i_, eliminating some potential sources provides new insights into the advantages of having a PP_i_-dependent glycolysis versus an ATP-dependent glycolysis and the trade-off between energy yield and thermodynamic driving force. Finally, our findings could help to predict the impact of changing from a PP_i_-dependent glycolysis to an ATP-dependent glycolysis and guide future metabolic engineering attempts aimed to increase the ethanol production capacity of *C. thermocellum*.

## MATERIALS AND METHODS

### Strains and maintenance.

All *C. thermocellum* strains used in this study (Table 4) originate from DSM1313 (Deutsche Sammlung von Mikroorganismen und Zellkulturen GmbH, Braunschweig, Germany; GenBank accession number CP002416). Stock cultures were grown anaerobically in CTFUD medium (32). Escherichia coli strains used in this study (Table 4) originate from a BL21 derivative (New England Biolabs catalog number C2566I; purchased from BioNordika AB, Solna, Sweden). Stock cultures were propagated in LB medium (10 g L^−1^ peptone, 5 g L^−1^ yeast extract, 10 g L^−1^ NaCl) supplemented with 100 μg ml^−1^ ampicillin. Frozen stocks were prepared by the addition of glycerol (25%, vol/vol) to overnight cultures and stored in 1-ml aliquots in cryogenic vials (VWR International AB, Stockholm, Sweden) at −80°C. For *C. thermocellum*, stocking was done in a vinyl anaerobic chamber from Coy Laboratory Products with 5% H_2_, 10% CO_2_, and 85% N_2_ (Strandmöllen AB, Ljungby, Sweden).

**TABLE 4:** Strains used in this study

Strain name	Parental strain	Organism	Relevant genotype	Accession no.	Source or reference
E. coli T7 Express		E. coli	*fhuA2 lacZ*::*T7 gene1* [*lon*] *ompT gal sulA11 R*(*mcr-73*::*miniTn10-TetS*)*2* [*dcm*] *R*(*zgb-210*::*Tn10-TetS*) *endA1 Δ*(*mcrC-mrr*)*114*::*IS10*		New England Biolabs (C2566I; Ipswich, MA, USA)
BL21 pTrc99a	E. coli T7 Express	E. coli	E. coli T7 Express with empty plasmid control pTrc99a		This study
BL21 pTK30	E. coli T7 Express	E. coli	E. coli T7 Express with *ags1–ags2* (*clo1313_0717-0718*) expression plasmid pTK30		This study
BL21 pTK50	E. coli T7 Express	E. coli	E. coli T7 Express with *clo1313_0997* expression plasmid pTK50		This study
BL21 pTK51	E. coli T7 Express	E. coli	E. coli T7 Express with *T. saccharolyticum pfk* (*tsac_1362*) expression plasmid pTK51		This study
BL21 pTK52	E. coli T7 Express	E. coli	E. coli T7 Express with *clo1313_1832* expression plasmid pTK52		This study
BL21 pTK53	E. coli T7 Express	E. coli	E. coli T7 Express with *clo1313_2627* expression plasmid pTK53		This study
BL21 pTK54	E. coli T7 Express	E. coli	E. coli T7 Express with *clo1313_1686* expression plasmid pTK54		This study
BL21 pTK55	E. coli T7 Express	E. coli	E. coli T7 Express with E. coli *acs* (*b21_03901*) expression plasmid pTK55		This study
Wild-type or LL1004		*C. thermocellum*	Wild-type DSM1313	CP002416	DSMZ
LL1041 or M1448	LL345 or M1354	*C. thermocellum*	DSM1313 Δ*hpt* Δ*pta*	SRP054855	34
AVM003	LL1004	*C. thermocellum*	LL1004 Δ*ppdk* (*clo1313_0949*)	SAMN20219718	This study
AVM008	LL1004	*C. thermocellum*	LL1004 Δ*ppase* (*clo1313_0823*)	SAMN20219719	This study
AVM051	LL1004	*C. thermocellum*	LL1004 Δ*P_ags1_*_,_*_2_-ags1 ags2* (P*_clo1313_0717-0718_*-*clo1313_0717-0718*)	SAMN20219720	This study
AVM059	LL1004	*C. thermocellum*	LL1004 Δ*clo1313_1686*	SAMN20219721	This study
AVM052	AVM003	*C. thermocellum*	LL1004 Δ*ppdk* Δ*P_ags1_*_,_*_2_-ags1 ags2*	SAMN20219722	This study
AVM053	AVM008	*C. thermocellum*	LL1004 Δ*ppase* Δ*P_ags1_*_,_*_2_-ags1 ags2*	SAMN20219723	This study
AVM060	AVM051	*C. thermocellum*	LL1004 Δ*P_ags1_*_,_*_2_-ags1 ags2* Δ*clo1313_1686*	SAMN20219724	This study
AVM056	AVM053	*C. thermocellum*	LL1004 Δ*ppase* Δ*P_ags1_*_,_*_2_-ags1 ags2* Δ*ppdk*	SAMN20219725	This study
AVM061	AVM056	*C. thermocellum*	LL1004 Δ*ppase* Δ*P_ags1_*_,_*_2_-ags1 ags2* Δ*ppdk* Δ*clo1313_1686*	SAMN20219726	This study

### Plasmid construction.

All plasmids used in this study are listed in Table 5. Deletion plasmids were *in vitro* assembled with the Gibson assembly protocol (70, 71) using the pDGO145 backbone, P*_gapDH_*-*cat*-*hpt* cassette, and the regions homologous to the 5′-upstream, 3′-downstream, and internal region of the genes of interest as cloning fragments (32). For the Gibson assembly, 0.15 pmol DNA of the 5′-flank, 3′-flank, and internal region fragments was mixed with 0.03 pmol of pDGO145 backbone and P*_gapDH_*-*cat*-*hpt* cassette. For the E. coli expression plasmids, Gibson assembly was used with 0.03 pmol pTrc99a backbone and 0.15 pmol coding sequence of the gene(s) of interest. DNA fragments were PCR amplified from pDGO145 or pTrc99a or from genomic DNA of *C. thermocellum* LL1004 (wild-type), Thermoanaerobacterium saccharolyticum JW/SL-YS 485 (DSM8691; GenBank accession number CP003184), or an E. coli BL21 derivate using Phusion high-fidelity DNA polymerase (Thermo Fisher Scientific, Waltham, MA, USA) according to the manufacturer´s instructions with primers ordered from Thermo Fisher Scientific or Integrated DNA Technologies (IDT; Skokie, IL, USA) (Table 6). For the deletion plasmids, primers were designed that add at least 30-bp homologous overhangs to the 5′ and 3′ ends of the 5′-flank, 3′-flank, and internal region cloning fragments. For the expression plasmids, at least 30-bp overhangs were added to the 5′ and 3′ ends of the coding sequence of the gene(s) of interest. Genomic DNA, plasmid DNA, and PCR products were purified using commercially available kits from GeneJET (Thermo Fisher Scientific), with the exception of genomic DNA from *T. saccharolyticum* JW/SL-YS 485, which was purchased directly from DSMZ. After Gibson assembly, 20 μl of Gibson reaction mix was used to transform 50 μl of E. coli BL21 derivative cells (catalog number C2566I; New England Biolabs). E. coli BL21 cells were made chemically competent using rubidium chloride (72). Correct plasmid assembly was confirmed via diagnostic PCR and Sanger sequencing (Eurofins Genomics Sweden AB, Solna, Sweden) of the open reading frames, homologous flanks, and promoters. Diagnostic PCR was performed on plasmid DNA isolated from randomly picked E. coli colonies using DreamTaq DNA polymerase (Thermo Fisher Scientific).

**TABLE 5:** Plasmids used in this study

Plasmid name	Relevant characteristic(s)	Accession no.	Source or reference
pDGO145	Deletion vector backbone	KY852359	81
pLL1228	*ppase* (*clo1313_0823*) markerless deletion vector	MT415065	18
pSH226	*clo1313_1686* markerless deletion vector	MZ502412	This study
pTK3	*ppdk* (*clo1313_0949*) markerless deletion vector	MZ502413	This study
pTK20	*P_ags1_*_,_*_2_-ags1 ags2* (P*_clo1313_0717-0718_*-*clo1313_0717-0718*) markerless deletion vector; Int region homologous to internal region of *clo1313_0717*	MZ502414	This study
pTK22	*P_ags1_*_,_*_2_-ags1 ags2* (P*_clo1313_0717-0718_*-*clo1313_0717-0718*) markerless deletion vector; Int region homologous to internal region of *clo1313_0718*	MZ502415	This study
pTrc99a	E. coli high-expression vector (empty vector control)	U13872	Pharmacia Biotech (Uppsala, Sweden)
pTK30	pTrc99a with *ags1–ags2* (*clo1313_0717-0718*) expressed from the inducible *trc* promoter	MZ502416	This study
pTK50	pTrc99a with *clo1313_0997* expressed from the inducible *trc* promoter	MZ502417	This study
pTK51	pTrc99a with *T. saccharolyticum pfk* (*tsac_1362*) expressed from the inducible *trc* promoter	MZ502418	This study
pTK52	pTrc99a with *clo1313_1832* expressed from the inducible *trc* promoter	MZ502419	This study
pTK53	pTrc99a with *clo1313_2627* expressed from the inducible *trc* promoter	MZ502420	This study
pTK54	pTrc99a with *clo1313_1686* expressed from the inducible *trc* promoter	MZ502421	This study
pTK55	pTrc99a with E. coli *acs* (*b21_03901*) expressed from the inducible *trc* promoter	MZ502422	This study

**TABLE 6:** Primers used in this study

No.	Purpose	Sequence^*a*^ (5′ to 3′)
224	Amplification of pDGO145 backbone	GATATCGCCTCGTGATACGC
225	Amplification of pDGO145 backbone	CAGCTGCTAATAGTAGTGAAAAAATCAG
63	Amplification of P*_gapDH_*-*cat*-*hpt* selection cassette from pDGO145	GTGGGAATAGGCATGGAAAAGATTTTTTTGCC
64	Amplification of P*_gapDH_*-*cat*-*hpt* selection cassette from pDGO145	GGGGAGGGCGTGAATGTAAGCGTGA
222	Amplification of 5′-flanking region of *clo1313_0949* for pTK3	tattatcatgacattaacctataaaaataggcgtatcacgaggcgatatcGCATTTTGCCGTTATGTGCC
100	Amplification of 5′-flanking region of *clo1313_0949* for pTK3	ccttattatttctgtcccaaatcctttgtaccCCTTTTCCTCCAAGCAGGTC
101	Amplification of 3′-flanking region of *clo1313_0949* for pTK3	gcatcaatgagagacctgcttggaggaaaaggGGTACAAAGGATTTGGGACAG
102	Amplification of 3′-flanking region of *clo1313_0949* for pTK3	cgggcaaaaaaatcttttccatgcctattcccacCCCTCACCCTTGCTTCATATG
105	Amplification of internal region of *clo1313_0949* for pTK3	gttatgtcacgcttacattcacgccctccccAGTTTGTGGAGATAGCCGAAAAAC
223	Amplification of internal region of *clo1313_0949* for pTK3	ttcggttagagcggcattatccctgattttttcactactattagcagctgGGCAACGCAGCAAGTACCCA
419	Amplification of 5′-flanking region of P*_clo1313_0717-0718_*-*clo1313_0717-0718* for pTK20 and pTK22	ataaaaataggcgtatcacgaggcgatatcGTCCATACCGGAGGAAAAGC
420	Amplification of 5′-flanking region of P*_clo1313_0717-0718_*-*clo1313_0717-0718* for pTK20 and pTK22	cataatatcaaccttctttatctcttgcaaaaacCACCTGTTAATTTACATTTATCCGCC
418	Amplification of 3′-flanking region of P*_clo1313_0717-0718_*-*clo1313_0717-0718* for pTK20 and pTK22	tataggcggataaatgtaaattaacaggtgGTTTTTGCAAGAGATAAAGAAGG
395	Amplification of 3′-flanking region of P*_clo1313_0717-0718_*-*clo1313_0717-0718* for pTK20 and pTK22	gcaaaaaaatcttttccatgcctattcccacAGGTTCTTTTTAAGCTCGCC
390	Amplification of internal region of *clo1313_0717* for pTK20	ttatgtcacgcttacattcacgccctccccCATGTTTACAAGATGAACTATTCCC
417	Amplification of internal region of *clo1313_0717* for pTK20	ctgattttttcactactattagcagctgTTCCTGACACTGCCGTATATC
396	Amplification of internal region of *clo1313_0718* for pTK22	ttatgtcacgcttacattcacgccctccccGTGCCGATGCCATGTACCATAAC
397	Amplification of internal region of *clo1313_0718* for pTK22	ccctgattttttcactactattagcagctgGGGATTCAGCATCTCCATGTTAATC
XSH0987	Amplification of 5′-flanking region of *clo1313_1686* for pSH226	taggcgtatcacgaggcgatGAACTGGGCATTGACAGC
XSH0988	Amplification of 5′-flanking region of *clo1313_1686* for pSH226	tttgaagttgTATATAAAACCTCCATTATAAAATTATAGCC
XSH0989	Amplification of 3′-flanking region of *clo1313_1686* for pSH226	ggaggttttatataCAACTTCAAAATAACAACTTGCAAAATAAATG
XSH0990	Amplification of 3′-flanking region of *clo1313_1686* for pSH226	tccatgcctattcccacgatCTTTGAAATCTTCGGCATTGC
XSH0991	Amplification of internal region of *clo1313_1686* for pSH226	tacctggcccagtagttcagGTTATGGCAAAAGCTCTTATCG
XSH0992	Amplification of internal region of *clo1313_1686* for pSH226	tttttcactactattagcagTTCAGGATTCAGTGGTTCACC
482	Amplification of pTrc99a backbone for pTK30, pTK50–pTK55	GGCTGTTTTGGCGGATGAGA
483	Amplification of pTrc99a backbone for pTK30	attagtcctccttattcgggtacgtctgattaggCATGGTCTGTTTCCTGTGTG
617	Amplification of pTrc99a backbone for pTK50–pTK55	CATGGTCTGTTTCCTGTGTG
484	Amplification of *clo1313_0717-0718* genes for pTK30	cctaatcagacgtacccgaataaggaggactaatATGCATAAAAAGGAGATTATTGCCTTGCTG
485	Amplification of *clo1313_0717-0718* genes for pTK30	gaaaatcttctctcatccgccaaaacagccTTATATTACCGCATTTTTTCCTATTGTTATAGG
618	Amplification of *clo1313_0997* for pTK50	gataacaatttcacacaggaaacagaccatgATGAGCAGTGTAAGAACGATTG
619	Amplification of *clo1313_0997* for pTK50	gaaaatcttctctcatccgccaaaacagccTTATAATGCTAATATTTTGCTAAGCTGAATC
620	Amplification of *T. saccharolyticum pfk* (*tsac_1362*) for pTK51	gataacaatttcacacaggaaacagaccatgATGAGAACAATAGGAGTTTTAACAAGTGGTG
621	Amplification of *T. saccharolyticum pfk* (*tsac_1362*) for pTK51	gaaaatcttctctcatccgccaaaacagccTTAAATTGATAATATTTTGCTGAGTTCATAAAGC
622	Amplification of *clo1313_1832* for pTK52	gataacaatttcacacaggaaacagaccatgATGTTTGATGTTGTTGCGGTTG
623	Amplification of *clo1313_1832* for pTK52	gaaaatcttctctcatccgccaaaacagccTTACTTCTCTTCAAGAAACTGCCTC
624	Amplification of *clo1313_2627* for pTK53	gataacaatttcacacaggaaacagaccatgATGATAACATCTGTGGCTCTC
625	Amplification of *clo1313_2627* for pTK53	gaaaatcttctctcatccgccaaaacagccTCAAGATATTCTCTCAATTTCCACTCTG
632	Amplification of *clo1313_1686* for pTK54	gataacaatttcacacaggaaacagaccatgATGTTAGAGAAATATTTGTCAAAAGTAAATTTTAATTC
633	Amplification of *clo1313_1686* for pTK54	gaaaatcttctctcatccgccaaaacagccTTATTTTTTTGCCTGGTCCTTGTTTCTG
634	Amplification of E. coli *acs* (*b21_03901*) for pTK55	gataacaatttcacacaggaaacagaccatgATGAGCCAAATTCACAAACACACC
635	Amplification of E. coli *acs* (*b21_03901*) for pTK55	gaaaatcttctctcatccgccaaaacagccTTACGATGGCATCGCGATAG
282	Confirmation of correct deletion plasmid assembly	GCCACCTGACGTCTAAGAAA
281	Confirmation of correct deletion plasmid assembly	AAGAAAACAGACGCGCCC
280	Confirmation of correct deletion plasmid assembly	GGAACCTTCCTTTTATAGGCG
284	Confirmation of correct deletion plasmid assembly	GTTAGAGCGGCATTATCCCT
157	Confirmation of correct deletion plasmid assembly	GGCAGCTAATAGAGGCATTA
156	Confirmation of correct deletion plasmid assembly	CCTAACTCTCCGTCGCTATT
163	Confirmation of correct deletion plasmid assembly	CCTGATGAATGAGTTGAGCTTC
248	Confirmation of correct plasmid assembly pLL1228	CTGAGGGCACGCAGTTTAGG
249	Confirmation of correct plasmid assembly pLL1228	GCTTCCGGTATTTAGTCAGGTGC
524	Confirmation of correct plasmid assembly pSH226	CTCGAACCGGATGACATGAC
525	Confirmation of correct plasmid assembly pSH226	CCATTCCTTGTCACCGTTGAAC
246	Confirmation of correct plasmid assembly pTK3	GCAATGCATCAATGAGAGACCTGC
247	Confirmation of correct plasmid assembly pTK3	GTTATTGCTTTTTCCTGCTACCAAACAC
406	Confirmation of correct plasmid assembly pTK20 and pTK22	CATAATCCTGTAAGGCTGAAGC
329	Confirmation of correct plasmid assembly pTK20 and pTK22	AGCGGAGTTTTGGAGTACGTC
486	Confirmation of correct expression plasmid assembly	CTGTGCAGGTCGTAAATCACTG
628	Confirmation of correct expression plasmid assembly	CGGCGTTTCACTTCTGAGTTC
325	Confirmation of correct plasmid assembly pTK30	CATAGCATACAGGGATATGAGC
401	Confirmation of correct plasmid assembly pTK30	TGAGCAAATGGTTGACTCAG
404	Confirmation of correct plasmid assembly pTK30	GAATCAGCCCATGACTTCGG
626	Confirmation of correct plasmid assembly pTK50	CTCGATGGCTTTGCCTTCTC
627	Confirmation of correct plasmid assembly pTK50	CAGAGGAGCAAGAGACATCAG
327	Confirmation of correct plasmid assembly pTK51	GCACTATCGACAATGACATACCG
330	Confirmation of correct plasmid assembly pTK51	CCAGTACAATTATGTGATGCAGC
139	Confirmation of correct plasmid assembly pTK52	GTGCGGATGTCAGTTTGGAC
629	Confirmation of correct plasmid assembly pTK52	GCCAAAGTTCTTCTTCACTGAC
630	Confirmation of correct plasmid assembly pTK53	GAGACAGTGAGACGGAAGTAC
631	Confirmation of correct plasmid assembly pTK53	GCGCCTCAACAATTTCGTCC
636	Confirmation of correct plasmid assembly pTK54	TGGCGGTTACAATATGGCCC
637	Confirmation of correct plasmid assembly pTK54	AGGATATGGTCAGACAGAGC
638	Confirmation of correct plasmid assembly pTK55	CCAGTAGATATCACCCGGATG
639	Confirmation of correct plasmid assembly pTK55	CGTACTGGCGGGAAAATTGAC
640	Confirmation of correct plasmid assembly pTK55	TAAGCGTGACGTAGGCGTAG
641	Confirmation of correct plasmid assembly pTK55	CAACGAGAAATGTCCGGTGG
33	Confirmation of P*_gapDH_*-*cat*-*hpt* selection cassette removal; confirmation of correct deletion plasmid assembly	GCTATCTTTACAGGTACATCATTCTGTTTGTG
34	Confirmation of P*_gapDH_*-*cat*-*hpt* selection cassette removal	TTTCATCAAAGTCCAATCCATAACCC
285	Confirmation of P*_cbp_*-*tdk* selection marker removal	ACGTTATATTGCTTGCCGGG
289	Confirmation of P*_cbp_*-*tdk* selection marker removal	AAGACTCCTTTGCTCCAACC
146	Confirmation of *clo1313_0949* deletion	GTTTCCGGCTATACCCAACG
147	Confirmation of *clo1313_0949* deletion	CGTTTCAGGGTCAACAGCCA
148	Confirmation of *clo1313_0949* deletion	GCGATGTTGTCATGGAGGTG
149	Confirmation of *clo1313_0949* deletion	GATCCAGGAATCTGATCGTCAC
423	Confirmation of P*_clo1313_0717-0718_*-*clo1313_0717-0718* deletion	CGACAATTACGGAGAGATTGAG
326	Confirmation of P*_clo1313_0717-0718_*-*clo1313_0717-0718* deletion	CATAAGCCCGTCATCGTAAAC
398	Confirmation of P*_clo1313_0717-0718_*-*clo1313_0717-0718* deletion	CGATATTGATACGGTAGGAGTGC
313	Confirmation of P*_clo1313_0717-0718_*-*clo1313_0717-0718* deletion; Confirmation of correct plasmid assembly pTK30	GCTCCCTCACTTACATAAACACC
399	Confirmation of P*_clo1313_0717-0718_*-*clo1313_0717-0718* deletion	GTCGAATATGGTAAACTCCGGC
400	Confirmation of P*_clo1313_0717-0718_*-*clo1313_0717-0718* deletion; confirmation of correct plasmid assembly pTK30	GTGACTCCGCGGAAAAGTAC
242	Confirmation of *clo1313_0823* deletion	GCAATGCGGAACTGGTGAAGGC
243	Confirmation of *clo1313_0823* deletion	CATCCATCGCAAACACGGCATGG
244	Confirmation of *clo1313_0823* deletion	GAGCGTTTTTGTCAACACCCAGC
245	Confirmation of *clo1313_0823* deletion	GGTACGGTTCTTTCAGCACTGGC
495	Confirmation of *clo1313_1686* deletion	CAGAAAGGATGGTTCCATGTC
496	Confirmation of *clo1313_1686* deletion	GGAGATTTCAGAAGCCCTTG
497	Confirmation of *clo1313_1686* deletion; confirmation of correct plasmid assembly pTK54	CTCGCTCTTCACAAGATAGG
498	Confirmation of *clo1313_1686* deletion; confirmation of correct plasmid assembly pTK54	CAAGGATTGCCATTTTCGTCAAG
1	Amplification of 16S rRNA fragment for culture purity confirmation	AGAGTTTGATCCTGGCTCAG
2	Amplification of 16S rRNA fragment for culture purity confirmation	ACGGCTACCTTGTTACGACTT

a Uppercase letters indicate the primer annealing sequences. Lowercase letters indicate the ≥30-bp overhang sequences used for Gibson assembly.

### Strain construction.

Transformations and gene deletions of *C. thermocellum* were performed using previously described methods (32). H^+^-pumping membrane-bound pyrophosphatase (*clo1313_0823*; *ppase*) was deleted in the wild-type strain LL1004 using plasmid pLL1228, resulting in strain AVM008. Pyruvate phosphate dikinase (*clo1313_0949*; *ppdk*) was deleted in LL1004 using plasmid pTK3, yielding strain AVM003. The putative acetyl-CoA synthetase (*clo1313_1686*) was deleted in LL1004 using plasmid pSH226, resulting in strain AVM059. ADP-glucose synthase (P*_clo1313_0717-0718_-clo1313_0717-0718*; *P_ags1_*_,_*_2_*-*ags1 ags2*) was deleted in LL1004 using plasmid pTK22, yielding strain AVM051. The double mutant strains, AVM052 and AVM053, were obtained by deletion of *P_ags1_*_,_*_2_*-*ags1 ags2* in strain AVM003 (Δ*ppdk*) or strain AVM008 (Δ*ppase*) using plasmid pTK22 or pTK20, respectively. The third double mutant strain, AVM060, was constructed by deletion of *clo1313_1686* in strain AVM051 (ΔP*_ags1_*_,_*_2_*-*ags1 ags2*) using plasmid pSH226. The triple mutant strain AVM056 was obtained by deletion of *ppdk* using plasmid pTK3 in strain AVM053 (Δ*ppase* ΔP*_ags1_*_,_*_2_*-*ags1 ags2*). Finally, deletion of *clo1313_1686* in strain AVM056 (Δ*ppase* Δ*P_ags1_*_,_*_2_-ags1 ags2* Δ*ppdk*) using plasmid pSH226 yielded the quadruple mutant strain AVM061.

All E. coli protein expression strains were constructed by transforming 20 μl of the respective Gibson assembly plasmid mix to 50 μl chemically competent BL21 derivative cells (New England Biolabs), yielding strains BL21 pTK30, BL21 pTK50, BL21 pTK51, BL21 pTK52, BL21 pTK53, BL21 pTK54, and BL21 pTK55. BL21 pTrc99a was constructed by transforming ca. 150 ng of purified pTrc99a plasmid to 50 μl chemically competent BL21 derivative cells (New England Biolabs).

Genetic modifications for *C. thermocellum* were confirmed via diagnostic PCR with DreamTaq DNA polymerase (Thermo Fisher Scientific), using primer combinations binding outside the targeted loci as well as inside the targeted loci (Table 6). Additionally, Sanger sequencing (Eurofins Genomics Sweden AB) of the modified loci and whole-genome sequencing were used to confirm gene deletions. Culture purity of constructed strains was routinely checked through Sanger sequencing (Eurofins Genomics Sweden AB) with 16S rRNA primers from IDT (Table 6).

### Media and cultivation.

Serum bottle cultures were grown in 125-ml Wheaton serum bottles (DWK Life Sciences, Millville, NJ, USA) containing 50 ml modified low-carbon (LC) medium (30, 39) with 5 g L^−1^ cellobiose as a carbon source. Serum bottles were sealed with blue butyl rubber stoppers (Chemglass Life Sciences, Vineland, NJ, USA) and aluminum crimp caps (Sigma-Aldrich, St. Louis, MO, USA). LC medium was prepared from sterile stock solutions as described by Yayo et al. (30), with minor modifications. Solution B, containing Na_2_SO_4_, KH_2_PO_4_, and K_2_HPO_4_, was concentrated 1.15-fold. Solution A (20-fold concentrated cellobiose), solution C (50-fold concentrated urea), solution D (50-fold concentrated MgCl_2_, CaCl_2_, FeSO_4_, FeCl_2_, and l-cysteine HCl), solution E (50-fold concentrated vitamins), and solution TE (1,000-fold concentrated trace elements) were added to solution B to reach the final concentrations. After adding all medium components, bottles were purged for 5 cycles (45 s per cycle) with a gas mix of 20% CO_2_ and 80% N_2_ prior to inoculation to initiate growth.

Initial preculture serum bottles were inoculated from frozen glycerol stocks (−80°C). After overnight incubation, these initial precultures were transferred in mid-exponential phase (optical density at 600 nm [OD_600_], 0.5 to 1.0) to fresh precultures. Serum bottles were inoculated from these exponentially growing fresh preculture serum bottles (OD_600_, 0.5 to 1.5) to an initial OD_600_ of 0.05. These serum bottles were sampled throughout the exponential growth phase (up to an OD_600_ of 2.5) for OD_600_ and extracellular metabolite analysis (as described below). All serum bottle cultures were incubated at 55°C in a Jeio Tech ISS-4075R incubator shaker (Milmedtek AB, Karlskrona, Sweden) set at 180 rpm.

### Growth arrest studies.

For growth arrest experiments, the washed-cell experimental method of Zhou et al. (9) was used, with some modifications. Growth arrest was achieved with two different media based on either nitrogen depletion (as used by Zhou et al. [9]) or sulfur depletion. For both methods, cells were harvested from exponentially growing cultures (OD_600_, 1.2 to 1.8) grown on 100 ml modified LC medium (30, 39) in 200-ml Kimble serum bottles (DWK Life Sciences). Upon harvesting, cells were transferred in the anaerobic chamber to two 50-ml Falcon tubes sealed with anaerobic vinyl tape (TG Instrument AB, Helsingborg, Sweden) and centrifuged in an Avanti J-20 XP centrifuge (Beckman Coulter, Brea, CA, USA) at 6,500 × *g* for 15 min at 4°C. Cell pellets were resuspended in 1.8 ml modified LC medium without cellobiose, cysteine, and Na_2_SO_4_ (for S depletion) or in 1.8 ml modified LC medium without cellobiose and urea (for N depletion), transferred to a 2-ml microtube (Sarstedt AB, Helsingborg, Sweden), centrifuged (6,500 × *g*, 12 min, 25°C) in a table-top centrifuge (Microstar 12; VWR), and washed twice with the same modified LC medium. Washed cells were used to inoculate 125-ml Wheaton serum bottles (DWK Life Sciences) containing 50 ml of one of five modified LC media, i.e., LC medium, LC medium without Na_2_SO_4_ and with 10-fold lower cysteine levels (0.01 g L^−1^) (for S-depletion), LC medium without cellobiose and Na_2_SO_4_ but with 0.01 g L^−1^ cysteine (S-depletion control), LC medium without urea (for N-depletion), and LC medium without cellobiose and urea (N-depletion control). All washes and inoculation were done under anaerobic conditions. Modified LC media were purged extensively for 20 cycles (45 s per cycle) with pure N_2_ gas, directly followed by purging for 5 cycles (45 s per cycle) with a gas mix of 20% CO_2_ and 80% N_2_, and immediately transferred to the anaerobic chamber to ensure complete anaerobic conditions. After inoculation, bottles were sampled regularly over the course of 24 to 72 h for both OD_600_ and extracellular metabolite analysis as described below.

### CDW and optical density determination.

Cell dry weight (CDW) measurements were performed in technical triplicate by adding 10 ml of culture sample to predried and preweighed conical glass tubes and centrifuging (2,250 × *g*, 20 min) the tubes in a table-top centrifuge (Z206 A; Hermle Labortechnik GmbH, Wehingen, Germany). Cell pellets were washed once with deionized water and dried overnight in a forced-convection drying oven (VENTI-Line; VWR International AB) at 105°C. CDW was determined by dividing the dry weight of the cells by the volume of the culture sample. Optical density was measured in technical triplicate at 600 nm in a V-1200 spectrophotometer (VWR International AB). For calculation of the biomass yield on cellobiose, CDW was estimated from OD_600_ measurements using a conversion factor of 2.6. This factor was determined from the slope of OD_600_ against CDW based on a calibration curve of five samples. The calibration curve was made by serial dilution of culture samples taken at an OD_600_ of 2.0 (OD_600_ values after dilution: 0.5, 0.8, 1.0, 1.5, and 2.0) for strain LL1004 (wild type), AVM003 (Δ*ppdk*), AVM051 (Δ*P_ags1_*_,_*_2_-ags1 ags2*), AVM056 (Δ*ppase* Δ*P_ags1_*_,_*_2_-ags1 ags2* Δ*ppdk*), and AVM061 (Δ*ppase* Δ*P_ags1_*_,_*_2_-ags1 ags2* Δ*ppdk* Δ*clo1313_1686*) and was consistent between all strains.

### Extracellular metabolite analysis.

Culture supernatants were obtained by centrifuging 0.6 ml culture sample in a table-top centrifuge (Centrifuge 5424; Eppendorf, Hamburg, Germany) at 20,238 × *g* for 2 min. After initial centrifugation, the supernatant was filtrated with Corning Costar Spin-X centrifuge tube filters (0.22-μm nylon membrane; Sigma-Aldrich) at 20,238 × *g* for 2 min. Filtered supernatant was stored for up to 1 week at 4°C until further analysis. Extracellular concentrations of acetate, ethanol, formate, lactate, pyruvate, malate, glucose, and cellobiose were determined in culture supernatants on a Waters Alliance 2695 high-performance liquid chromatography (HPLC) (Waters, Milford, MA, USA) containing a Bio-Rad Aminex HPX-87H column (Bio-Rad, Hercules, CA, USA). The HPLC was operated with 5 mM H_2_SO_4_ as the mobile phase at a flow rate of 0.6 ml min^−1^ at 60°C. Pyruvate, malate, and formate were detected with 75 mM H_2_SO_4_ as the mobile phase. Detection was done with a Waters 2996 photodiode array detector at 210 nm and a Waters 2414 refractive-index detector. The column was heated with a Waters temperature control module.

### Glycogen assay.

Glycogen content was measured in technical triplicate by adding 1 ml of culture sample to 5 ml ice-cold methanol (−80°C) and centrifuging (10,000 × *g*, 10 min, −10°C) in an Avanti J-20 XP centrifuge (Beckman Coulter). The supernatant was decanted and the cell pellet was dissolved in 1.8 ml ice-cold methanol, transferred to a 2-ml microtube (Sarstedt AB), and centrifuged (10,000 × *g*, 10 min, 4°C) in a table-top centrifuge. The cell pellet was stored at −80°C until further analysis. After thawing the cell pellets on ice, glycogen was analyzed as described previously (73). Glucose released from glycogen conversion was measured by HPLC as described above.

### Calculation of yields and specific growth rate.

Yields on cellobiose (in mol mol^−1^ or g*_x_* g^−1^) and maximum specific growth rates (μ^max^; per hour) of each fermentation were determined from at least five samples taken during the exponential growth phase (OD_600_, 0.4 to 2.2). Yields on cellobiose were calculated by plotting the product concentrations against the cellobiose concentration and using the absolute slopes of the resulting linear fit made by linear regression. The maximum specific growth rate during exponential growth was calculated from the slope of the semilogarithmic plot of OD_600_ against time. All fermentation data can be found in File S4 in the supplemental material.

### Protein expression in E. coli.

For high-level expression of genes of interest from the *trc* promoter in E. coli, 500 μl of an overnight culture was inoculated into 500-ml baffled shake flasks containing 100 ml LB medium supplemented with 100 μg ml^−1^ ampicillin. Cultures were grown aerobically at 37°C in an orbital shaker (Infors, Basel, Switzerland) set at 180 rpm. At an OD_600_ of 0.6, protein expression was induced by addition of isopropyl-β-d-1-thiogalactopyranoside at a final concentration of 200 μM. After 4 h of incubation, cells were harvested, centrifuged (6,500 × *g*, 15 min, 4°C) in an Avanti J-20 XP centrifuge (Beckman Coulter), washed twice with 100 mM cold Tris-HCl buffer (pH 7.5 at 25°C), and stored at −20°C until further use. BL21 pTrc99a was used as an empty vector control strain.

### SDS-PAGE.

To verify expression of genes of interest in E. coli, SDS-PAGE gels were run. Three volumes of cell extract samples were mixed with one volume of 4× NuPAGE LDS sample buffer (Thermo Fisher Scientific) and incubated at 105°C for 15 min. For each cell extract sample, approximately 6 μg of protein solution (in 10 to 15 μl) and 10 μl of SeeBlue Plus2 prestained protein standard (Thermo Fisher Scientific) were loaded into wells of a NuPAGE 10% Bis-Tris gel (Thermo Fisher Scientific). Gel electrophoresis was run at 180 V for 60 min in morpholinepropanesulfonic acid (MOPS) running buffer (10.46 g L^−1^ MOPS, 6.06 g L^−1^ Tris, 1 g L^−1^ SDS, 0.3 g L^−1^ EDTA) at room temperature. After electrophoresis, gels were stained with PageBlue protein staining solution (Thermo Fisher Scientific) according to the manufacturer’s instructions.

### Preparation of cell extracts for *in vitro* enzyme activity assays.

*C. thermocellum* cell extracts for *in vitro* measurement of pyruvate phosphate dikinase, acetyl-CoA synthetase, lactate dehydrogenase, ADP-glucose synthase, and phosphofructokinase activity were prepared as described earlier (30).

Prior to the enzyme activity assays of ADP-glucose synthase, cell extracts were dialyzed against 500 ml of 100 mM cold Tris-HCl buffer (pH 7.5 at 25°C) with 2 mM 1,4-dithiothreitol (DTT) and 10 mM MgCl_2_ for 2 h at 4°C using 3-ml 10,000 molecular weight cutoff Slide-A-Lyzer dialysis cassettes (Thermo Fisher Scientific).

For *in vitro* enzyme activity assays of the membrane-bound pyrophosphatase, *C. thermocellum* cells were harvested as described above, with minor modifications. After harvesting the cells, the cell pellets were washed and resuspended in 10 mM MOPS-tetramethylammonium (TMA) hydroxide buffer (pH 7.2 at 25°C) prior to storage at −20°C. To prepare cell extracts, cell suspensions were thawed on ice, centrifuged (6,500 × *g*, 15 min, 4°C) in an Avanti J-20 XP centrifuge (Beckman Coulter), and resuspended in 10 mM MOPS-TMAOH buffer (pH 7.2 at 25°C) with 5 mM MgCl_2_, 5 mM DTT, 50 μM EGTA, and one tablet of cOmplete protease inhibitor cocktail (Sigma-Aldrich). Cells were disrupted by triple passage through a prewashed and prechilled FA-078 SLM Aminco French press (SLM Instruments Inc., Urbana, IL, USA) at 19,200 lb/in^2^. Between each passage, cell suspensions were cooled on ice for 5 min. Whole cells and cell debris were removed by centrifugation (20,000 × *g*, 30 min, 4°C).

E. coli cell extracts were prepared by thawing the cell suspension on ice, centrifuging (6,500 × *g*, 15 min, 4°C), and resuspending the cell pellet in 100 mM Tris-HCl buffer (pH 7.5 at 25°C) with 2 mM DTT and 10 mM MgCl_2_. Cells were lysed by single passage through a prewashed and prechilled FA-078 SLM Aminco French press (SLM Instruments Inc.) at 12,800 lb/in^2^. The cell extract was obtained by centrifuging the cell lysate at 15,000 × *g* for 20 min at 4°C.

For phosphofructokinase enzyme activity assays with E. coli strains BL21 pTK50, BL21 pTK51, BL21 pTK52, and BL21 pTK53, the cell extract was split in two fractions. One fraction was used directly in the assays, whereas the other fraction was heat treated for 30 min at 60°C to remove native E. coli background activity (as described previously [49]). The heat-treated sample was subsequently centrifuged at 15,000 × *g* for 20 min at 4°C to remove precipitated proteins.

All cell extracts were stored on ice and used on the same day.

### *In vitro* enzyme activity assays.

Enzyme activity assays were performed aerobically with fresh cell extracts at 37°C or 55°C with a Cary 50 UV-visible spectrophotometer equipped with a single-cell Peltier element (Varian AB, Solna, Sweden). All assays were done in quartz cuvettes (Sigma-Aldrich) with 1-cm path length and 1- or 3-ml reaction mixtures. Enzyme activities are reported in μmol · min^−1^ · (mg protein)^−1^ and are averages from biological duplicate measurements. For each biological replicate, two concentrations of cell extract were assayed in technical duplicate to confirm proportionality between the enzyme activity and the added amount of cell extract.

To ensure that all enzyme activity assays were performed at exactly 37°C or 55°C, all reaction components except for coupling enzymes (if needed), cell extract, and the substrate used to start the reaction were added as concentrated stock solutions to preheated Milli-Q (at 37°C or 55°C) and incubated for 2 or 5 min at 37°C or 55°C, respectively. After this interval, coupling enzymes and the cell extract were added and incubated for 2 or 4 min at 37°C or 55°C, respectively. The reaction was subsequently started by adding the starting substrate as described below.

Activities of pyruvate phosphate dikinase (EC 2.7.9.1) and lactate dehydrogenase (EC 1.1.1.27) were determined as previously described by Olson et al. (8) and Lo et al. (74), respectively. Lactate dehydrogenase was routinely assayed as a quality check of the cell extract (Table S15).

Acetyl-CoA synthetase (EC 6.2.1.1) was assayed as described by van den Berg et al. (33), with minor modifications. The reaction mixture (1 ml) contained 100 mM Tris-HCl (pH 7.5), 10 mM l-malate disodium salt, 0.2 mM coenzyme A, 8 mM ATP, 1 mM NAD^+^, 10 mM MgCl_2_, 18 U of l-malate dehydrogenase (from pig heart; 10127256001; Sigma-Aldrich), 3.3 U of citrate synthase (from porcine heart; C3260; Sigma-Aldrich), and 50 or 100 μl cell extract. The reaction was started by addition of 100 mM potassium acetate.

ADP-glucose synthase (EC 2.7.7.27) was assayed in the pyrophosphorolysis direction according to Plaxton and Preiss (75), with minor modifications. The reaction mixture (1 ml) contained 50 mM Tris-HCl (pH 7.5), 4 mM MgCl_2_, 2 mM fructose-1,6-bisphosphate, 0.2 g L^−1^ bovine serum albumin, 1 mM ADP-, UDP-, or GDP-glucose, 0.6 mM NADP^+^, 0.01 mM glucose-1,6-bisphosphate, 4 mM potassium fluoride, 1.8 U of phosphoglucomutase (from rabbit muscle, P3397; Sigma-Aldrich), 2.2 U of glucose-6-phosphate dehydrogenase (from Saccharomyces cerevisiae, G7877; Sigma-Aldrich), and 50 or 100 μl cell extract. The reaction was started by addition of 1 mM K_4_PP_i_. For ADP-glucose synthase assays with *C. thermocellum* cell extracts, several optimizations to this protocol were attempted, which are described below.

ATP- and PP_i_-dependent phosphofructokinase (EC 2.7.1.11 or EC 2.7.1.90) was assayed according to Zhou et al. (9), with some modifications. The reaction mixture (1 ml) contained 50 mM Tris-HCl (pH 7.0), 5 mM MgCl_2_, 0.15 mM NADH, 1 mM fructose-6-phosphate, 4 U of aldolase (from rabbit muscle, A8811; Sigma-Aldrich), 4 U of triosephosphate isomerase (from S. cerevisiae, T2507; Sigma-Aldrich), 4 U of α-glycerophosphate dehydrogenase (from rabbit muscle, G6751; Sigma-Aldrich), and 50 or 100 μl cell extract. The reaction was started by addition of 2 mM ATP or PP_i_. For GTP-dependent phosphofructokinase assays, 2 mM GTP was added at the start and incubated for 2 min with 2 U of inorganic pyrophosphatase (from yeast, 10108987001; Roche) to remove PP_i_ impurities in the GTP stock. The reaction was started by addition of 1 mM fructose-6-phosphate. In addition to these standard conditions, the following variations were attempted for the ATP- and GTP-dependent Pfk assays: (i) use of 50 mM imidazole-HCl (pH 7.0 at 55°C) or MOPS (pH 7.0 at 55°C) as assay buffer, (ii) dialysis of the cell extract as described above, and (iii) addition of various concentrations of ATP or GTP (0.05 to 8 mM) and fructose-6-phosphate (0.25 to 20 mM).

The fructokinase (EC 2.7.1.4) assay was adapted from Yayo et al. (30) and contained 50 mM Tris-HCl (pH 7.5), 5 mM MgCl_2_, 120 mM KCl, 2 mM fructose, 2 mM NADP^+^, 2 U of glucose-6-phosphate isomerase (from S. cerevisiae, P5381; Sigma-Aldrich), 2.2 U of glucose-6-phosphate dehydrogenase (from S. cerevisiae, G7877; Sigma-Aldrich), and 50 or 100 μl cell extract. The reaction was started by addition of 2 mM ATP or GTP.

Membrane-bound pyrophosphatase (EC 7.1.3.1) was discontinuously assayed based on hydrolysis of pyrophosphate (PP_i_) to inorganic phosphate (P_i_) (76). The assay mixture (3 ml) contained 100 mM MOPS-TMAOH (pH 7.2), 5.3 mM MgCl_2_, 40 μM EGTA, 10 mM NaCl, 50 mM KCl, and 20, 40, 50, 100, or 200 μl cell extract. The reaction was started by addition of 160 μM K_4_PP_i_. After 10 s and 5 min, a 550-μl sample was taken and stored immediately on ice. Liberated inorganic phosphate was detected using a malachite green detection assay (77). A volume of 40 μl of malachite green color reagent was mixed with 160 μl of sample in triplicate in a flat-bottom 96-well plate (Eppendorf Cell Culture Plates; Sigma-Aldrich) and incubated at room temperature for 25 min. After exactly 25 min, the absorbance was measured at 630 nm in a plate reader (AH Diagnostics AB, Solna, Sweden). Phosphate concentrations were calculated from *A*_630_ values using a phosphate calibration curve (range, 0 to 30 μM P_i_). Enzyme activities were corrected for acid-catalyzed hydrolysis of PP_i_, which was determined by omitting cell extract from the assay mixture.

Protein concentrations in cell extracts were quantified according to Bradford (78) with bovine serum albumin (Sigma-Aldrich) as the standard.

### Optimizations of ADP-glucose synthase assay.

Since ADP-glucose synthase activity assays with *C. thermocellum* cell extracts did not show any activity with the above-described protocol, several modifications to this protocol were attempted both in the preparation of the cell extract as well as in the assay conditions used. For the preparation of the cell extract, we (i) harvested and prepared the cell(-free) extracts in 100 mM MOPS (pH 7.5 at 25°C), HEPES (pH 7.5 at 25°C), or potassium-phosphate buffer (pH 7.5 at 25°C) instead of Tris-HCl buffer (pH 7.5 at 25°C) as described above, (ii) added one tablet of cOmplete protease inhibitor cocktail (Sigma-Aldrich) to the lysis buffer, (iii) performed the harvest and preparation of the cell(-free) extracts under anaerobic conditions, and (iv) dialyzed the cell extract prior to the *in vitro* enzyme activity measurement according to the protocol described above. The assay modifications included (i) the use of 50 mM MOPS, HEPES, or potassium-phosphate buffer at a (ii) pH of 7.0, 7.5, or 8.0, (iii) varying the assay temperature to 37, 45, and 55°C, and (iv) assaying the activity under anaerobic conditions.

### Whole-genome sequencing.

Whole-genome resequencing for all strains constructed in this study was used to verify strain construction and check for secondary mutations, as described before (79).

### (i) Library preparation.

Genomic DNA was prepared using the Omega E.Z.N.A. kit by following the manufacturer’s protocol (Omega Bio-Tek, GA, USA). A volume of 500 ng of DNA was used for next-generation sequencing (NGS) library preparation using the NEBNext Ultra II FS DNA library prep kit for Illumina (New England Biolabs, MA, USA). Fractionated, adapter-ligated DNA fragments went through 5 rounds of PCR amplification and purification. The resulting NGS library was sequenced at the Genomics and Molecular Biology Shared Resource (GMBSR) at Dartmouth.

### (ii) Sequencing.

Libraries were diluted to 4 nM, pooled, and loaded at 1.8 pM onto a NextSeq500 Mid Output flow cell, targeting 130 million 2 × 150-bp reads/sample. Base-calling was performed on instrument using RTA2 and bcls converted to fastq files using bcl2fastq2 v2.20.0.422.

### (iii) Data analysis.

Read data were analyzed with the CLC Genomic Workbench, version 12 (Qiagen Inc., Hilden, Germany). Reads were mapped to the reference genome (GenBank accession no. NC_017992) with an average read coverage of at least 45-fold. Mapping was improved by two rounds of local realignment. The CLC Basic Variant Detection algorithm was used to determine small mutations (single- and multiple-nucleotide polymorphisms, short insertions, and short deletions). Variants occurring in less than 35% of the reads or fewer than 4 reads were filtered out. The fraction of the reads containing the mutation is presented in File S2. To determine larger mutations, the CLC InDel and Structural Variant algorithm was run. This tool analyzes unaligned ends of reads and annotates regions where a structural variation may have occurred, which are called breakpoints. Since the read length averaged 150 bp and the minimum mapping fraction was 0.5, a breakpoint can have up to 75 bp of sequence data. The resulting breakpoints were filtered to eliminate those with fewer than 10 reads or less than 20% not perfectly matched. The breakpoint sequence was searched with the Basic Local Alignment Search Tool (BLAST) algorithm (80) for similarity to known sequences. Pairs of matching left and right breakpoints were considered evidence for structural variations, such as transposon insertions and gene deletions. The fraction of the reads supporting the mutation (left and right breakpoints averaged) is presented in File S2. Mutation data from CLC were further processed using custom Python scripts (https://github.com/danolson1/cth-mutation).

### optStoic procedure.

The optStoic algorithm (31) is used to probe for pathways capable of generating pyrophosphate via the net stoichiometric conversion ${\text{ATP}}_{\text{eq}}+{\text{P}}_{\text{i}}\to {\text{ADP}}_{\text{eq}}+{\text{PP}}_{\text{i}}$ using the reaction content of the most recent *C. thermocellum* genome-scale metabolic model (i.e., iCBI655 [26]) as well as the complete list of reactions present in the KEGG database (44) (7,164 reactions). The minFlux optimization formulation within optStoic minimizes the total reaction flux required to achieve the desired net stoichiometric conversion. An in-depth description of the optStoic algorithm and the minFlux optimization formulation can be found elsewhere (31). By successively excluding the previously obtained solutions and rerunning minFlux, a total of 100 distinct pathway designs were identified using reactions from only model iCBI655 and 500 with the full complement of KEGG reactions. From the set of 500 solutions generated using KEGG reactions, only solutions that contained at most one new reaction that was not present in the iCBI655 genome-scale metabolic model were retained.

### Data analysis.

Unpaired Student’s *t* test was used for comparison between values in this study.

### Data availability.

Whole-genome sequencing data of the engineered strains was deposited into the NCBI Sequence Read Archive (https://www.ncbi.nlm.nih.gov/sra) with accession numbers listed in Table 4. Plasmid sequence accession numbers are shown in Table 5 (MZ502412 to MZ502422).
