# Supplementary material for: Functional Analysis of H+-Pumping Membrane-Bound Pyrophosphatase, ADP-Glucose Synthase, and Pyruvate Phosphate Dikinase as Pyrophosphate Sources in Clostridium thermocellum
Source: Appl Environ Microbiol. 2022 Feb 22;88(4):e01857-21. doi: 10.1128/aem.01857-21 (PMC8863071; doi:10.1128/aem.01857-21)
Supplement: Supplemental file 5 — Tables S1 to S15, Fig. S1 to S4. Download aem.01857-21-s0005.pdf, PDF file, 0.5 MB [file aem.01857-21-s0005.pdf]

SUPPLEMENTARY FILES

Table S1. Pyrophosphate stoichiometry of precursor metabolites formed from cellobiose. Central building blocks produced from the precursor metabolites are indicated.

**Precursor metabolites**	**PP_i formed (+) or consumed (-) in mol per mol precursor metabolite**	**Central building blocks produced from precursor metabolites**
Glucose-6-phosphate	0.00	Lipids, lipoteichoic acids, and cell wall components
Fructose-6-phosphate	0.00	None
Ribose-5-phosphate[a]	-0.17	Histidine, tryptophan, and nucleotides
Erythrose-4-phosphate[a]	-0.33	Phenylalanine, tryptophan, and tyrosine
Glyceraldehyde-3-phosphate	-0.50	Lipids, lipoteichoic acids, and cell wall components
3-phosphoglycerate	-0.50	Cysteine, glycine, and serine
Phosphoenolpyruvate	-0.50	Phenylalanine, tyrosine and tryptophan
Oxaloacetate	-0.50	Asparagine, aspartate, lysine, methionine, threonine, nucleotides
Pyruvate[b]	-0.50 – x	Alanine, isoleucine, leucine, lysine, and valine

Acetyl-CoA[b]	-0.50 – x	Isoleucine, leucine, lipids, lipoteichoic acids, and cell wall components
α-ketoglutarate[b]	-1 – x	Arginine, glutamate, glutamine, and proline
Succinyl-CoA[b]	-1 – x	None

[a]PP_i stoichiometry based on non-oxidative pentose-phosphate pathway as proposed by Koendjbiharie et al. (1).

[b]Parameter x describes the flux distribution between the Ppdk reaction and the malate shunt. If x = 1, the PEP-to-pyruvate conversion solely goes through Ppdk. If x = 0, the PEP-to-pyruvate conversion solely goes through the malate shunt.

4

Table S2. Pyrophosphate stoichiometry of amino acids formed from cellobiose.

Amino acid	PP_i formed (+) or consumed (-) in mol per mol amino acid[a,b]	Amino acid composition (% w/w)[c]	Amino acid stoichiometry (mmol per g cells)	PP_i stoichiometry (mmol per g cells)
Alanine	-0.50 – x	6.38	0.299	-0.150 – 0.299x
Arginine[d]	-x	4.37	0.205	-0.205x
Asparagine	+0.50	5.39	0.253	+0.126
Aspartate	-0.50	5.69	0.267	-0.133
Cysteine[d]	+0.50	1.17	0.055	+0.027
Glutamate	-1 – x	7.62	0.357	-0.357 – 0.357x
Glutamine	-1 – x	2.57	0.120	-0.120 – 0.120x
Glycine[d]	-0.50	6.68	0.313	-0.157
Histidine	+1.50	1.43	0.067	+0.100
Isoleucine	-1.50 – 3x	8.49	0.398	-0.597 – 1.193x
Leucine	-1.50 – 3x	8.66	0.406	-0.609 – 1.217x
Lysine	-1 – x	8.10	0.380	-0.380 – 0.380x
Methionine[d]	+0.50	2.51	0.118	+0.059
Phenylalanine	-1.33	4.24	0.199	-0.265
Proline	-1 – x	3.39	0.159	-0.159 – 0.159x
Serine	-0.50	6.04	0.283	-0.142

Threonine	-0.50	5.04	0.236	-0.118
Tryptophan[e]	+x	0.89	0.042	+0.042x
Tyrosine	-1.33	4.23	0.198	-0.264
Valine	-1 – 2x	7.11	0.333	-0.333 – 0.667x
**Total amino acids**				**-3.471 – 4.556x**
Polymerization				+4.688
**Total amino acids including polymerization**				**1.218 – 4.556x**

[a]Amino acids are assumed to be formed through the most used biochemical pathways as used in the iCBI655 genome-scale metabolic model of *C. thermocellum* (2).

[b]For amino acids derived from pyruvate, acetyl-CoA or α-ketoglutarate, a degree of freedom (x) is included. This parameter describes the flux distribution between the Ppdk reaction and the malate shunt. If x = 1, the PEP-to-pyruvate conversion solely goes through Ppdk. If x = 0, the PEP-to-pyruvate conversion solely goes through the malate shunt.

[c]Composition is based on the amino acid counts in all open reading frames of the ATCC 27405 genome.

[d]PP_i stoichiometry was corrected for by-product formation of fumarate, acetate, or formate during the biosynthetic reactions by subtracting the PP_i stoichiometry of the closest precursor metabolite, i.e. oxaloacetate and acetyl-CoA, that can be derived from this by-product.

[e]PP_i stoichiometry was corrected for by-product formation of pyruvate during the biosynthetic reactions.

6

Table S3. Pyrophosphate stoichiometry of deoxynucleotides formed from cellobiose.

**Deoxynucleotide (dNMP)**	**PP_i formed (+) or consumed (-) in mol per mol dNMP[a]**	**DNA composition (mole dNMP per mole DNA)[b]**	**dNMP stoichiometry (mmol per g cells)**	**PP_i stoichiometry (mmol per g cells)**
dAMP[c]	+0.333	0.305	0.024	+0.008
dCMP	+0.333	0.196	0.016	+0.005
dGMP[c]	+1.333	0.196	0.016	+0.021
dTMP	+1.333	0.305	0.024	+0.033
**Total DNA precursors**				**+0.067**
Polymerization				+0.080
**Total DNA precursors including polymerization**				**+0.147**

[a]Deoxynucleotides are assumed to be formed through the most used biochemical pathways as used in the iCBI655 genome-scale metabolic model of *C. thermocellum* (2).

[b]Composition is based on the DSM 1313 genomic content.

[c]PP_i stoichiometry was corrected for by-product formation of fumarate during the biosynthetic reactions by subtracting the PP_i stoichiometry of the closest precursor metabolite, i.e. oxaloacetate, that can be derived from this by-product.

Table S4. Pyrophosphate stoichiometry of nucleotides formed from cellobiose.

Nucleotides (NMP)	PP_i formed (+) or consumed (-) in mol per mol NMP[a]	RNA composition (mole NMP per mole RNA)[b]	NMP stoichiometry (mmol per g cells)	PP_i stoichiometry (mmol per g cells)
AMP[c]	+0.333	0.305	0.059	+0.020
CMP	+0.333	0.196	0.038	+0.013
GMP[c]	+1.333	0.194	0.038	+0.050
UMP	+0.333	0.306	0.060	+0.020
**Total RNA precursors**				**+0.103**
Polymerization				+0.195
**Total RNA precursors including polymerization**				**+0.298**

[a]Nucleotides are assumed to be formed through the most used biochemical pathways as used in the iCBI655 genome-scale metabolic model of *C. thermocellum* (2).

[b]Composition is based on the genomic content of the open reading frames of the ATCC 27405 genome.

[c]PP_i stoichiometry was corrected for by-product formation of fumarate during the biosynthetic reactions by subtracting the PP_i stoichiometry of the closest precursor metabolite, i.e. oxaloacetate, that can be derived from this by-product.

Table S5. Pyrophosphate stoichiometry of fatty acids formed from cellobiose.

**Fatty acids**	**PP_i formed (+) or consumed (-) in mol per mol fatty acid[a,b]**	**Normalized fatty acid composition (% w/w)[c]**	**Normalized fatty acid composition (mol fatty acid per mol average fatty acid)**	**PP_i stoichiometry (mol per mol average fatty acid)**
C10:0 branched	-2.5 – 5x	7.43	0.051	-0.128 – 0.255x
C13:0 branched	-3.5 – 7x	2.61	0.022	-0.078 – 0.156x
C14:0 total[d]	-3.5 – 7x	5.92	0.054	-0.189 – 0.378x
C16:0 total[e]	-4 – 8x	53.92	0.552	-2.209 – 4.417x
C17:0 branched (anteiso)	-4.5 – 9x	12.35	0.133	-0.600 – 1.201x
C16:1	-4 – 8x	12.35	0.125	-0.502 – 1.004x
C18:0 total[f]	-4.5 – 9x	5.42	0.062	-0.277 – 0.555x
**Average fatty acid chain[g]**				**-3.983 – 7.966x**

[a]Fatty acids are assumed to be formed through the most used biochemical pathways as used in the iCBI655 genome-scale metabolic model of *C. thermocellum* (2).

[b]All fatty acids are derived from acetyl-CoA, hence, the degree of freedom (x) is included. This parameter describes the flux distribution between the Ppdk reaction and the malate shunt. If x = 1, the PEP-to-pyruvate conversion solely goes through Ppdk. If x = 0, the PEP-to-pyruvate conversion solely goes through the malate shunt.

[c]Fatty acid composition taken from Herrero et al. (3) for ATCC 27405. Composition is normalized to a total of 100%.

[d]C14:0 total is the sum of the C14:0 branched (iso), C14:0 normal, and C14:1 composition given by Herrero et al. (3).

[e]C16:0 total is the sum of the C16:0 branched (iso) and C14:0 normal composition given by Herrero et al. (3).

[f]C18:0 total is the sum of the C18:0 branched (iso) and C18:0 normal composition given by Herrero et al. (3).

[g]PP_i stoichiometry of the average fatty acid chain is used for the PP_i stoichiometry of lipids and lipoteichoic acids.

Table S6. Pyrophosphate stoichiometry of lipids formed from cellobiose.

Lipid	PP_i formed (+) or consumed (-) in mol per mol lipid[a,b]	Lipid composition (% w/w)[c]	Lipid stoichiometry (mmol per g cells)	PP_i stoichiometry (mmol per g cells)
Monoglucosyldiacylglycerol	-7.466 – 15.931x	8.11	0.009	-0.064 – 0.136x
Diglucosyldiacylglycerol	-6.466 – 15.931x	12.81	0.011	-0.071 – 0.175x
Triglucosyldiacylglycerol	-5.466 – 15.941x	9.11	0.007	-0.036 – 0.105x
Cardiolipin	-15.431 – 31.863x	0.80	0.001	-0.007 – 0.014x
Phosphatidylglycerol	-7.966 – 15.931x	16.52	0.018	-0.140 – 0.280x
Lysylphosphatidylglycerol	-7.966 – 16.931x	2.40	0.002	-0.017 – 0.037x
Phosphatidylethanolamine	-7.966 – 15.931x	50.25	0.056	-0.445 – 0.889x
**Total lipids**				**-0.779 – 1.636x**

[a]Lipids are assumed to be formed through the most used biochemical pathways as used in the iCBI655 genome-scale metabolic model of *C. thermocellum* (2).

[b]For lipids derived from pyruvate or acetyl-CoA, a degree of freedom (x) is included. This parameter describes the flux distribution between the Ppdk reaction and the malate shunt. If x = 1, the PEP-to-pyruvate conversion solely goes through Ppdk. If x = 0, the PEP-to-pyruvate conversion solely goes through the malate shunt.

[c]Composition taken from Matsumoto et al. (4) for aerobically grown *B. subtilis* cultures. Composition is normalized to a total of 100%. Phosphatidylserine (< 0.1%) and the underdetermined fraction of “others” (1.8%) were not considered. The fatty acid composition used to estimate the molecular weights of the lipids was taken from Herrero et al. (3) for ATCC 27405 (Table S5).

Table S7. Pyrophosphate stoichiometry of lipoteichoic acids formed from cellobiose.

Lipoteichoic acid (LTA)	PP_i formed (+) or consumed (-) in mol per mol LTA[a,b]	Lipoteichoic acid composition (% w/w)[c]	LTA stoichiometry (mmol per g cells)	PP_i stoichiometry (mmol per g cells)
Lipoteichoic acid (n=24), linked, glucose substituted	29.534 – 15.931x	19	0.001	0.020 – 0.011x
Lipoteichoic acid (n=24), linked, N-acetyl-D-glucosamine	17.534 – 39.931x	19	0.001	0.011 – 0.024x
Lipoteichoic acid (n=24), linked, D-alanine substituted	17.534 – 39.931x	40	0.002	0.034 – 0.077x
Lipoteichoic acid (n=24), linked, unsubstituted	5.534 – 15.931x	22	0.002	0.008 – 0.023x
**Total lipoteichoic acids**				**0.073 – 0.136x**

[a]Lipoteichoic acids are assumed to be formed through the most used biochemical pathways as used in the iCBI655 genome-scale metabolic model of *C. thermocellum* (2).

[b]For lipoteichoic acids derived from pyruvate or acetyl-CoA, a degree of freedom (x) is included. This parameter describes the flux distribution between the Ppdk reaction and the malate shunt. If x = 1, the PEP-to-pyruvate conversion solely goes through Ppdk. If x = 0, the PEP-to-pyruvate conversion solely goes through the malate shunt.

[c]Composition is taken from Dauner and Sauer (5) and Fischer (6) for *B. subtilis*. The fatty acid composition used to estimate the molecular weights of the lipoteichoic acids was taken from Herrero et al. (3) for ATCC 27405 (Table S5).

Table S8. Pyrophosphate stoichiometry of cell wall components formed from cellobiose.

Cell wall components (CWC)	PP_i formed (+) or consumed (-) in mol per mol CWC[a,b]	Cell wall composition (% w/w)[c]	CWC stoichiometry (mmol per g cells)	PP_i stoichiometry (mmol per g cells)
Peptidoglycan subunit	-3 – 7x	45	0.102	-0.305 – 0.711x
Glycerol teichoic acid (n=45), unlinked, unsubstituted	23.5 – 2x	11.9	0.004	0.085 – 0.007x
Glycerol teichoic acid (n=45), unlinked, D-alanine substituted	46 – 47x	11.9	0.003	0.116 – 0.118x
Glycerol teichoic acid (n=45), unlinked, glucose substituted	68.5 – 2x	11.9	0.002	0.124 – 0.004
Minor teichoic acid (acetylgalactosamine glucose phosphate, n=30)	45 – 30x	19.3	0.003	0.146 – 0.097x
**Total cell wall components**				**0.166 – 0.938x**

[a]Cell wall components are assumed to be formed through the most used biochemical pathways as used in the iCBI655 genome-scale metabolic model of *C. thermocellum* (2).

[b]For cell wall components derived from pyruvate or acetyl-CoA, a degree of freedom (x) is included. This parameter describes the flux distribution between the Ppdk reaction and the malate shunt. If x = 1, the PEP-to-pyruvate conversion solely goes through Ppdk. If x = 0, the PEP-to-pyruvate conversion solely goes through the malate shunt.

[c]Composition is taken from Dauner and Sauer (5) and Lang et al. (7) for *B. subtilis*.

Table S9. ADP-glucose synthase activities of cell-free extracts from *E. coli* BL21 pTrc99a and BL21 pTK30. Enzyme activity was assayed at 55 °C. Averages and standard deviations were obtained from two independent biological duplicates. The detection limit was 0.05 µmol mg protein^{-1} min^{-1}.

**Strain**	**Relevant genotype**	**Substrate**	**Enzyme activity (µmol mg protein^{-1} min^{-1})**
BL21 pTrc99a	Empty vector control	ADP-glucose	< 0.05
BL21 pTK30	*clo1313_0717-0718*↑[a] (*C. thermocellum*)	ADP-glucose	0.41 ± 0.06
		GDP-glucose	< 0.05
		UDP-glucose	< 0.05

[a]Upward pointing arrow indicates that the gene is overexpressed.

Table S10. Pyruvate and malate yields and carbon balance of *C. thermocellum* wild-type and mutant strains in batch serum bottle cultures. Cultures were grown on LC medium containing 5 g L^{-1} cellobiose. Yields and carbon balances were calculated from data obtained during the exponential growth phase. Averages and standard deviations were obtained from three independent biological replicates.

**Strain**	**Relevant genotype**	**Product yield (mol mol^{-1})**		**Carbon balance (%)**[a]
		**Pyruvate**	**Malate**	
LL1004	Wild-type	0.07 ± 0.00	0.01 ± 0.00	87.1 ± 2.7
AVM008	Δ*ppase*	0.09 ± 0.01	0.02 ± 0.00	85.1 ± 1.4
AVM051	Δ$P_{ags1,2}$-*ags1 ags2*	0.10 ± 0.01	0.01 ± 0.00	81.5 ± 1.4
AVM003	Δ*ppdk*	0.12 ± 0.00	0.02 ± 0.00	78.3 ± 0.9
AVM059	Δ*clo1313_1686*	0.08 ± 0.00	0.01 ± 0.00	89.6 ± 1.9
AVM053	Δ*ppase* Δ$P_{ags1,2}$-*ags1 ags2*	0.14 ± 0.03	0.01 ± 0.00	78.9 ± 2.7
AVM052	Δ*ppdk* Δ$P_{ags1,2}$-*ags1 ags2*	0.11 ± 0.00	0.02 ± 0.00	78.0 ± 2.7
AVM060	Δ$P_{ags1,2}$-*ags1 ags2* Δ*clo1313_1686*	0.12 ± 0.00	0.02 ± 0.01	86.5 ± 1.4
AVM056	Δ*ppase* Δ$P_{ags1,2}$-*ags1 ags2* Δ*ppdk*	0.11 ± 0.00	0.01 ± 0.00	81.0 ± 1.2
AVM061	Δ*ppase* Δ$P_{ags1,2}$-*ags1 ags2* Δ*ppdk* Δ*clo1313_1686*	0.09 ± 0.00	0.01 ± 0.00	74.4 ± 1.7

[a]The carbon balance includes CO_2 production which is estimated from the ethanol, acetate, formate, and malate yields as described previously (8).

Table S11. Acetyl-CoA synthetase activities of cell-free extracts from *C. thermocellum* wild-type and mutant strains. Averages and standard deviations were obtained from two independent biological duplicates. The detection limit was 0.05 µmol mg protein^{-1} min^{-1}.

**Strain**	**Relevant genotype**	**Enzyme activity (µmol mg protein^{-1} min^{-1})**
LL1041	Δ*hpt* Δ*pta*	< 0.05
LL1004	Wild-type	0.29 ± 0.04
AVM059	Δ*clo1313_1686*	0.35 ± 0.04
AVM056	Δ*ppase* Δ$P_{ags1,2}$-*ags1 ags2* Δ*ppdk*	0.36 ± 0.10
AVM061	Δ*ppase* Δ$P_{ags1,2}$-*ags1 ags2* Δ*ppdk* Δ*clo1313_1686*	0.30 ± 0.03

Table S12. Acetyl-CoA synthetase activities of cell-free extracts from *E. coli* BL21 pTrc99a, BL21 pTK54, and BL21 pTK55. Enzyme activity was assayed at 37 °C and 55 °C. Averages and standard deviations were obtained from two independent biological duplicates. The detection limit was 0.05 µmol mg protein^{-1} min^{-1}.

**Strain**	**Relevant genotype**		**Enzyme activity (µmol mg protein^{-1} min^{-1})**
BL21 pTrc99a	Empty vector control	37 °C	0.20 ± 0.03
		55 °C	< 0.05
BL21 pTK54	c*lo1313_1686*↑[a] (*C. thermocellum*)	37 °C	0.15 ± 0.03
		55 °C	< 0.05
BL21 pTK55	*b21_03901*↑ (*E. coli acs*)	37 °C	0.67 ± 0.08
		55 °C	< 0.05

[a]Upward pointing arrow indicates that the gene is overexpressed.

Table S13. ATP- and GTP-dependent phosphofructokinase activities of (heat-treated) cell-free extracts from *E. coli* BL21 pTrc99a, BL21 pTK50, BL21 pTK51, BL21 pTK52, and BL21 pTK53. Enzyme activity was assayed at 55 °C. Averages and standard deviations were obtained from two independent biological duplicates. The detection limit was 0.05 µmol mg protein^{-1} min^{-1}.

**Strain**	**Relevant genotype**		**Enzyme activity (µmol mg protein^{-1} min^{-1})**	
			**ATP**	**GTP**
BL21 pTrc99a	Empty vector control	Control	0.19 ± 0.05	0.15 ± 0.01
		Heat-treated	< 0.05	< 0.05
BL21 pTK50	c*lo1313_0997*↑[a] (*C. thermocellum*)	Control	0.20 ± 0.01	0.22 ± 0.03
		Heat-treated	< 0.05	< 0.05
BL21 pTK51	*tsac_1362*↑ (*T. saccharolyticum pfk*)	Control	1.05 ± 0.07	1.68 ± 0.15
		Heat-treated	3.58 ± 0.79	6.30 ± 0.31
BL21 pTK52	c*lo1313_1832*↑ (*C. thermocellum*)	Control	0.12 ± 0.03	0.09 ± 0.01
		Heat-treated	< 0.05	< 0.05
BL21 pTK53	c*lo1313_2627*↑ (*C. thermocellum*)	Control	0.11 ± 0.02	0.06 ± 0.02
		Heat-treated	< 0.05	< 0.05

[a]Upward pointing arrow indicates that the gene is overexpressed.

Table S14. Fructokinase activities of cell-free extracts from *E. coli* BL21 pTrc99a and BL21 pTK52. Enzyme activity was assayed at 55 °C. Averages and standard deviations were obtained from two independent biological duplicates. The detection limit was 0.05 µmol mg protein^{-1} min^{-1}.

**Strain**	**Relevant genotype**	**Substrate**	**Enzyme activity (µmol mg protein^{-1} min^{-1})**
BL21 pTrc99a	Empty vector control	ATP	< 0.05
		GTP	< 0.05
BL21 pTK52	c*lo1313_1832*↑[a] (*C. thermocellum*)	ATP	1.53 ± 0.01
		GTP	1.54 ± 0.33

[a]Upward pointing arrow indicates that the gene is overexpressed.

Table S15. Lactate dehydrogenase (Ldh) activities of cell-free extracts from *C. thermocellum* wild-type and mutant strains. Averages and standard deviations were obtained from two independent biological duplicates. Ldh activity is used as a quality control of the cell-free extract.

Strain	Relevant genotype	Enzyme activity (µmol mg protein^{-1} min^{-1})
LL1004[a]	Wild-type	0.67 ± 0.31
LL1004[b]	Wild-type	1.16 ± 0.07
AVM008[a]	Δ*ppase*	0.95 ± 0.01
AVM003[b]	Δ*ppdk*	0.78 ± 0.08
AVM056[b]	Δ*ppase* Δ$P_{ags1,2}$-*ags1 ags2* Δ*ppdk*	1.09 ± 0.17
AVM059[b]	Δ*clo1313_1686*	1.01 ± 0.26
AVM061[a]	Δ*ppase* Δ$P_{ags1,2}$-*ags1 ags2* Δ*ppdk* Δ*clo1313_1686*	0.85 ± 0.10
AVM061[b]	Δ*ppase* Δ$P_{ags1,2}$-*ags1 ags2* Δ*ppdk* Δ*clo1313_1686*	0.93 ± 0.06

[a]Cell-free extract obtained by disrupting cells with a French press (see methods).

[b]Cell-free extract obtained by lysing cells with lysozymes (see methods).

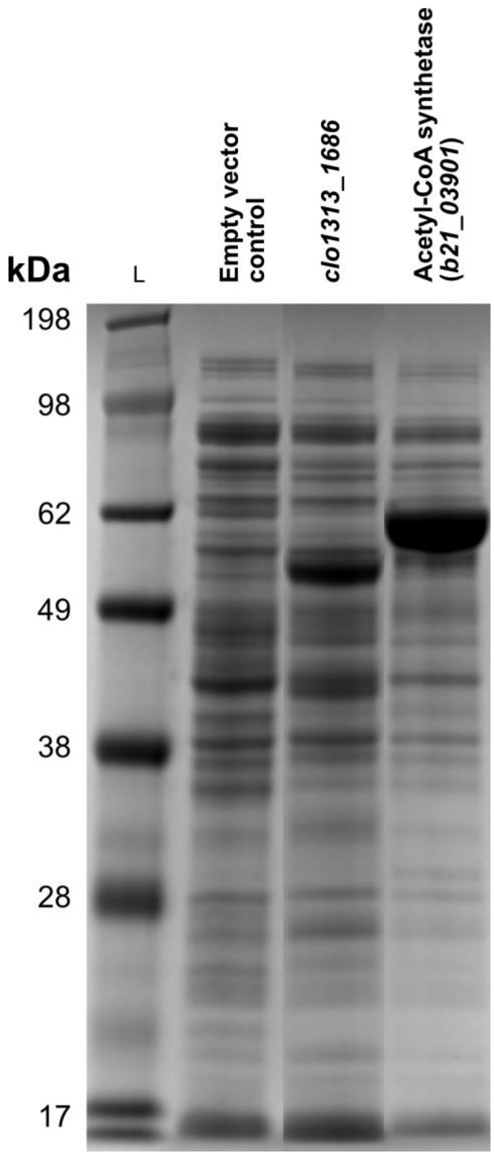

FIG S1. SDS-PAGE analysis of cell-free extracts of the *E. coli* strains BL21 pTrc99a (empty vector control), BL21 pTK54 (*clo1313_1686*↑), and BL21 pTK55 (*b21_03901*↑). The expected sizes are 63.1 kDa and 72.1 kDa for Clo1313_1686 and B21_03901 (*E. coli* Acs), respectively. Approximately 6 µg of protein solution was loaded per well. L denotes the SeeBlue™ Plus2 Pre-stained Protein Standard (Thermo Fisher Scientific); sizes are indicated.

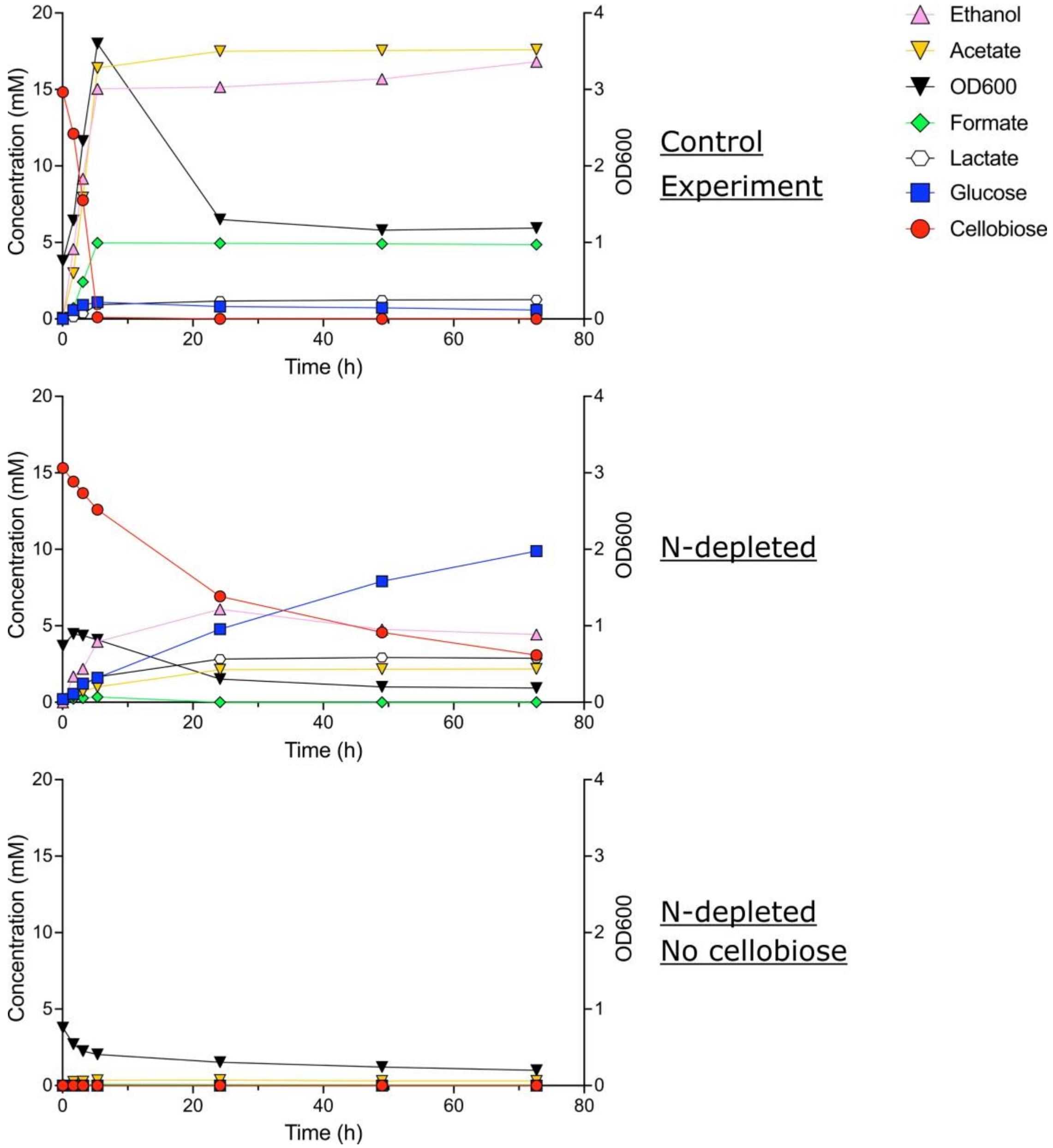

FIG S2. Growth and product profiles of LL1004 (wild-type) grown on LC medium (top panel; control experiment), LC medium without urea (middle panel; N-depleted), and LC medium without cellobiose and urea (bottom panel; N-depleted, no cellobiose). Data is shown for one representative experiment (*n* = 2).

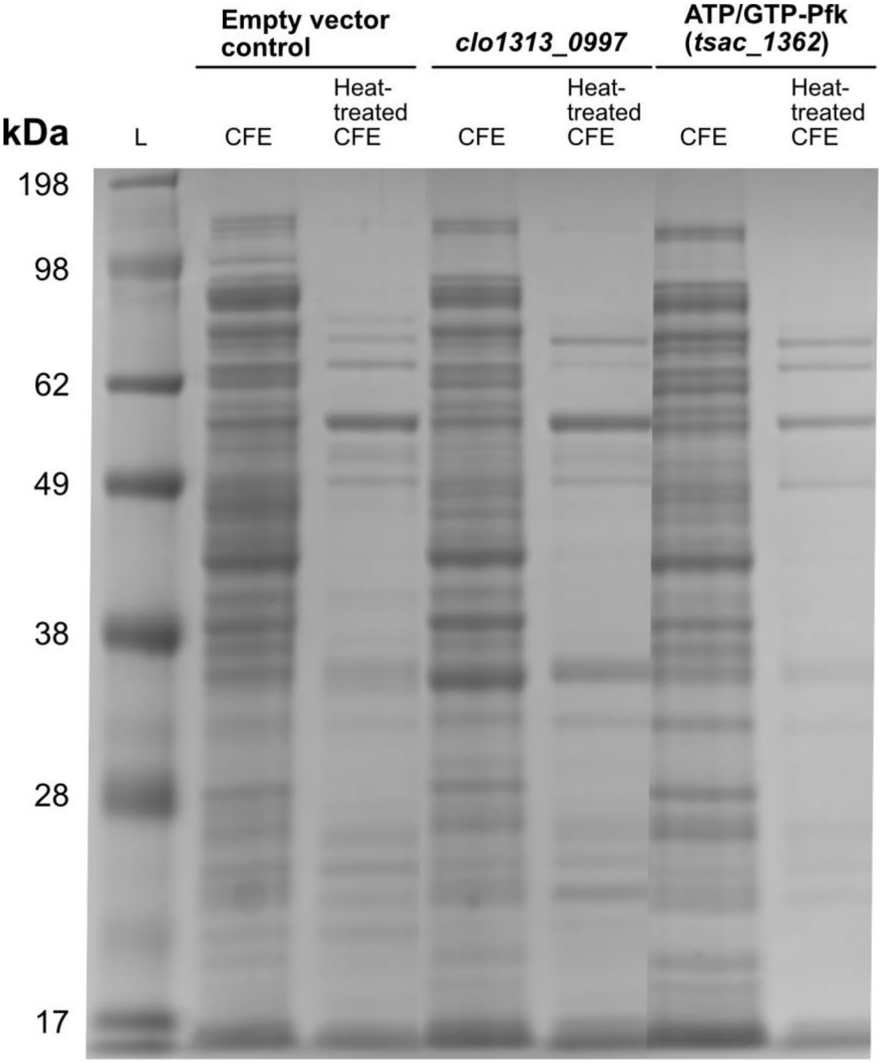

FIG S3. SDS-PAGE analysis of (heat-treated) cell-free extracts of the *E. coli* strains BL21 pTrc99a (empty vector control), BL21 pTK50 (*clo1313_0997*↑), and BL21 pTK51 (*tsac_1362*↑). The expected sizes are 34.8 kDa and 34.7 kDa for Clo1313_0997 and Tsac_1362 (*T. saccharolyticum* ATP/GTP-Pfk), respectively. Approximately 6 µg of protein solution was loaded per well. L denotes the SeeBlue™ Plus2 Pre-stained Protein Standard (Thermo Fisher Scientific); sizes are indicated.

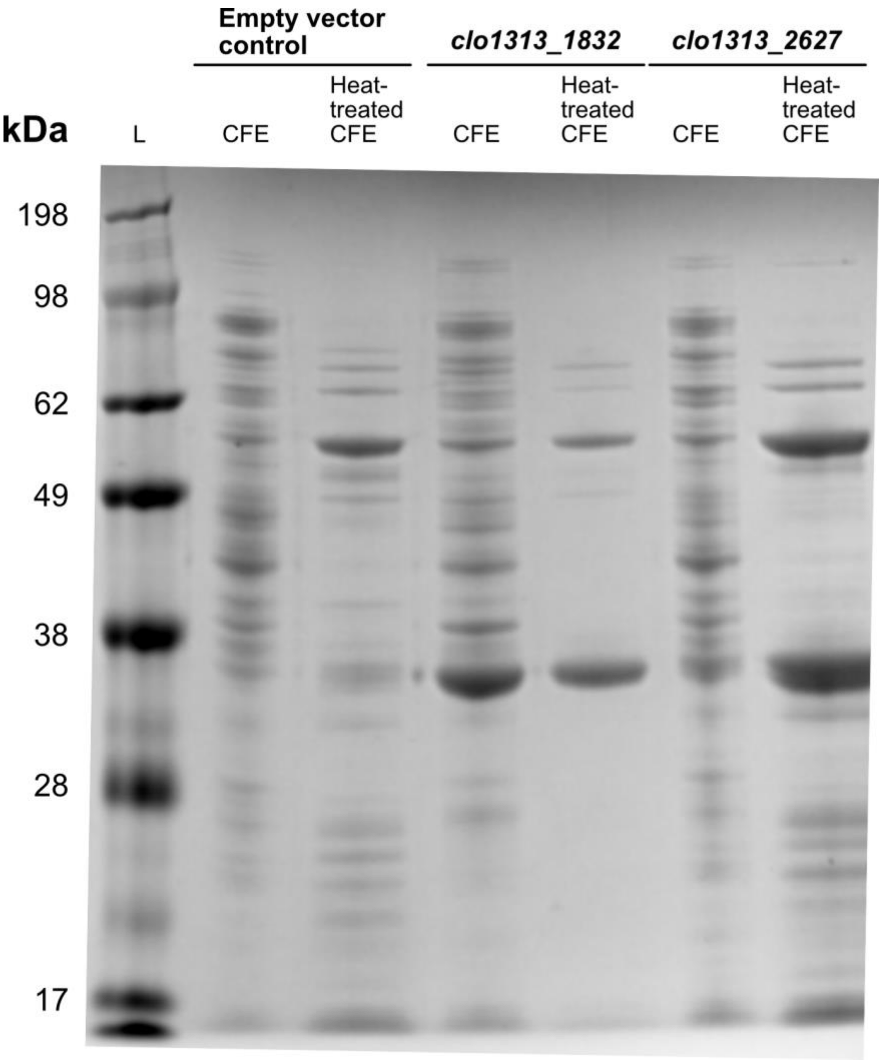

FIG S4. SDS-PAGE analysis of (heat-treated) cell-free extracts of the *E. coli* strains BL21 pTrc99a (empty vector control), BL21 pTK52 (*clo1313_1832*↑), and BL21 pTK53 (*clo1313_2627*↑). The expected sizes are 35.0 kDa and 33.8 kDa for Clo1313_1832 and Clo1313_2627, respectively. Approximately 6 µg of protein solution was loaded per well. L denotes the SeeBlue™ Plus2 Pre-stained Protein Standard (Thermo Fisher Scientific); sizes are indicated.

REFERENCES

1. Koendjbiharie JG, Hon S, Pabst M, Hooftman R, Stevenson DM, Cui J, Amador-Noguez D, Lynd LR, Olson DG, van Kranenburg R. 2020. The pentose phosphate pathway of cellulolytic clostridia relies on 6-phosphofructokinase instead of transaldolase. Journal of Biological Chemistry 295:1867-1878.
2. Garcia S, Thompson RA, Giannone RJ, Dash S, Maranas CD, Trinh CT. 2020. Development of a Genome-Scale Metabolic Model of *Clostridium thermocellum* and Its Applications for Integration of Multi-Omics Datasets and Computational Strain Design. Frontiers in Bioengineering and Biotechnology 8:772.
3. Herrero AA, Gomez RF, Roberts MF. 1982. Ethanol-induced changes in the membrane lipid composition of *Clostridium thermocellum*. Biochimica et Biophysica Acta (BBA) - Biomembranes 693:195-204.
4. Matsumoto K, Okada M, Horikoshi Y, Matsuzaki H, Kishi T, Itaya M, Shibuya I. 1998. Cloning, sequencing, and disruption of the *Bacillus subtilis psd* gene coding for phosphatidylserine decarboxylase. Journal of Bacteriology 180:100-106.
5. Dauner M, Sauer U. 2001. Stoichiometric growth model for riboflavin-producing *Bacillus subtilis*. Biotechnology and Bioengineering 76:132-143.
6. Fischer W. 1988. Physiology of Lipoteichoic Acids in Bacteria. Advances in Microbial Physiology 29:233-302.
7. Lang WK, Glassey K, Archibald AR. 1982. Influence of phosphate supply on teichoic acid and teichuronic acid content of *Bacillus subtilis* cell walls. Journal of Bacteriology 151:367-375.

8. Holwerda EK, Thorne PG, Olson DG, Amador-Noguez D, Engle NL, Tschaplinski TJ, van Dijken JP, Lynd LR. 2014. The exometabolome of *Clostridium thermocellum* reveals overflow metabolism at high cellulose loading. Biotechnology for Biofuels 7:155.
